# Bidirectional two-sample mendelian randomization analysis identifies causal associations of MRI-based cortical thickness and surface area relation to NAFLD

**DOI:** 10.1186/s12944-024-02043-x

**Published:** 2024-02-23

**Authors:** Zun Mao, Zhi-xiang Gao, Tong Ji, Sheng Huan, Guo-ping Yin, Long Chen

**Affiliations:** 1 Jiangsu Key Laboratory for Molecular and Medical Biotechnology, College of Life Sciences, Nanjing Normal University, Nanjing, 210023 P. R. China; 2 Department of Anesthesiology and Perioperative Medicine, the First Affiliated Hospital of Nanjing Medical University, Nanjing, 210000 P. R. China; 3 Department of Anesthesiology, Nanjing Second Hospital, Nanjing, 210000 P. R. China

**Keywords:** GWAS, NAFLD, Cortical structure, Brain-liver axis

## Abstract

**Background:**

Non-alcoholic fatty liver disease (NAFLD) patients have exhibited extra-hepatic neurological changes, but the causes and mechanisms remain unclear. This study investigates the causal effect of NAFLD on cortical structure through bidirectional two-sample Mendelian randomization analysis.

**Methods:**

Genetic data from 778,614 European individuals across four NAFLD studies were used to determine genetically predicted NAFLD. Abdominal MRI scans from 32,860 UK Biobank participants were utilized to evaluate genetically predicted liver fat and volume. Data from the ENIGMA Consortium, comprising 51,665 patients, were used to evaluate the associations between genetic susceptibility, NAFLD risk, liver fat, liver volume, and alterations in cortical thickness (TH) and surface area (SA). Inverse-variance weighted (IVW) estimation, Cochran Q, and MR-Egger were employed to assess heterogeneity and pleiotropy.

**Results:**

Overall, NAFLD did not significantly affect cortical SA or TH. However, potential associations were noted under global weighting, relating heightened NAFLD risk to reduced parahippocampal SA and decreased cortical TH in the caudal middle frontal, cuneus, lingual, and parstriangularis regions. Liver fat and volume also influenced the cortical structure of certain regions, although no Bonferroni-adjusted *p*-values reached significance. Two-step MR analysis revealed that liver fat, AST, and LDL levels mediated the impact of NAFLD on cortical structure. Multivariable MR analysis suggested that the impact of NAFLD on the cortical TH of lingual and parstriangularis was independent of BMI, obesity, hyperlipidemia, and diabetes.

**Conclusion:**

This study provides evidence that NAFLD causally influences the cortical structure of the brain, suggesting the existence of a liver-brain axis in the development of NAFLD.

**Supplementary Information:**

The online version contains supplementary material available at 10.1186/s12944-024-02043-x.

## Background

Non-alcoholic fatty liver disease (NAFLD), which accounts for a considerable percentage of chronic liver diseases worldwide and affects about 25% of the global population, is characterized by hepatic fat accumulation unrelated to excessive alcohol consumption [1–3]. This can incite inflammation, fibrosis, and ultimately leads to cirrhosis and hepatocellular carcinoma [1]. Numerous lines of investigation have suggested a relationship between NAFLD and extrapatic effects, namely neuropathological alterations, raising concerns over potential complications [4–7].

Patients with NAFLD demonstrate cognitive and neural characteristics similar to those seen in Alzheimer’s disease (AD), vascular dementia, and diabetes-associated cognitive decline [5–7]. NAFLD affects the central nervous system (CNS) via a variety of mechanisms, including metabolic disruptions, blood-brain barrier impairment, systemic inflammation, gut microbiota imbalances, and bile acid abnormalities [4, 8]. One study found specific dietary interventions with certain oils could regulate the expression of some genes to preserve neuron quantity and synaptic density in a porcine model of NAFLD [9]. Neurodegenerative diseases often involve neuronal loss and abnormalities in cortical structure, identifiable through T1-weighted MRI [10, 11]. Research indicated a significant association between NAFLD and reduced total brain volume, independent of confounders [6]. NAFLD animal models further corroborated this association [12]. However, linking NAFLD to cortical alterations has remained difficult owing to constraints in current research.

Individual risk for NAFLD and associated complications differs substantially, contingent on interplay between environmental exposures and polygenic host susceptibility factors [13]. Genome-wide association studies (GWAS) have uncovered robust and reproducible links between polymorphisms in PNPLA3, TM6SF2, MBOAT7, GCKR, HSD17B13 and NAFLD pathogenesis [14, 15]. Fine mapping of these genomic loci underscored the role of risk genes, such as MBOAT7, in cortical brain injury. MBOAT7, critical in murine neurodevelopment, may confer resilience against cortical and hippocampal decay alongside impeded neuron relocation [16]. Furthermore, PNPLA3 has been associated with carotid atherosclerosis and white matter hyperintensity (WMH) microbleeds, long considered important in the pathogenesis of cerebrovascular disease [17].

Neuropathological changes in NAFLD often precede clinical symptoms, such as AD or hepatic encephalopathy (HE) [6], suggesting that cortical structural changes could be an early indicator of hepatic dysfunction-related encephalopathy. Nevertheless, age-linked brain variations and comorbid conditions such as diabetes or obesity confound associations between NAFLD, neurodegeneration, and cortical architecture [18, 19]. Prior observational analyses sought to elucidate connections between cortical injury and NAFLD but were often constrained by aspects such as study design, sample size, and possible confounders [20, 21]. Mendelian Randomization (MR) provides an approach to surmount these barriers. This approach uses single nucleotide polymorphisms (SNPs) from GWAS as instrumental variables (IVs) to yield reliable causal inferences, assuming certain non-violable conditions are met [22]. Additionally, this technique precludes reverse causation since disease phenotypes do not impact genotypes. Previous MR studies, for example, have confirmed a potential causal association between NAFLD and stroke [23].

Here, we implement bidirectional two-sample MR to explore causal links between NAFLD and cortical structure. This was done to gain a deeper understanding of the etiology and progression of neurodegenerative changes induced by NAFLD.

## Methods

This study was conducted per STROBE-MR guidelines to ensure rigorous adherence to Mendelian randomization analysis protocol [24].

### Data sources for NAFLD, liver fat, and liver volume

A large pooled GWAS meta-analysis was conducted using data from over 778,000 individuals across four cohort studies to analyze genetic predictors of NAFLD risk [25]. The GWAS data was adjusted to account for potential confounding factors like age, gender, BMI, genotyping site, and ancestry. Data from GWAS on liver fat and liver volume were garnered from an earlier study involving abdominal MRI scans of 32,860 UK Biobank participants [26]. This GWAS data controlled for variables including age, sex, imaging center details, and genotyping batch. All participants involved in the aforementioned study were of European descent. NAFLD represents a physician-diagnosed chronic hepatic disorder. Hepatic lipid content, specifically liver fat, constitutes a primary characteristic and severity biomarker of NAFLD, with heterogeneity contingent on regional damage. Liver volume serves as a subtle but significant indicator of underlying liver damage, particularly reflecting the severity of liver fibrosis, tissue scarring, and fat accumulation [27, 28]. The challenge of accurately assessing NAFLD liver function stems from the complexity and multiplicity of liver functions. Accordingly, quantification of liver fat and volume proves critical for precise appraisal of NAFLD severity and hepatic functional effects.

### Data sources for cortical surface area (SA) and cortical thickness (TH)

Cortical architecture GWAS data were derived from the ENIGMA consortium [29] entailing MRI-based cortical TH and SA quantifications across 51,665 participants. Most data (approximately 94%) derived from 60 European ancestry cohorts (Supplementary Table 1). The Desikan-Killiany atlas provides the definition for the 34 regions, establishing a broad segmentation of the cortex where the boundaries of each region are determined by the gyrus anatomy marked in the depths of brain grooves. Each value represents the average of measurements taken from both hemispheres. Globally-weighted statistics capture region-specific cortical SA and TH measurements adjusted for total brain parameters. Conversely, non-globally weighted data represent region-restricted values without whole brain correction.

### Screening of genetic IVs

To determine the causal influence of NAFLD-related factors on cortical structure, we applied three groupings of genetic IVs capturing different facets of NAFLD hepatic pathobiology. These included: (i) index SNPs associated with NAFLD, (ii) index SNPs related to liver fat, and (iii) index SNPs pertaining to liver volume. All index SNPs related to exposure are enumerated in Supplementary Table 2. We clustered IV SNPs independently, utilizing the “TwoSampleM” package, with *P* < 5 × 10^− 8^. Stringent R^2^ < 0.001 and 1 MB window thresholds were instituted, with 1000 Genomes European data constituting the reference. When SNPs are in linkage disequilibrium, we retained those with the lowest *P* values. To eliminate the influence of other potential confounding factors on the study outcome variables, we used the Phenotype Scanner V2.0 database [30] to exclude SNPs associated with aging, cognitive impairment, organic brain changes, and mental disorders. After filtering out potential confounding SNPs, we applied Mendelian Randomization Pleiotropy RESidual Sum and Outlier (MR-PRESSO) to remove potential outliers before each MR analysis [31].

The F statistic appraises instrument strength dependent on the phenotypic variation attributable to genetics (R^2^), sample size (N) and number of variables (k). The F statistic is calculated using the following equation: F = R^2^(N − k−1)/k(1 − R^2^) [32]. To determine the R^2^ for the IV SNPs less than 10, this formula is applied: 2×EAF×(1 − EAF)×beta^2^. However, for the extended 10 IV SNPs, the following formula is used: (2×EAF×(1 − EAF)×beta^2^)/[(2×EAF×(1 − EAF)×beta^2^)+(2×EAF×(1 − EAF)×N×SE(beta^2^)] [33]. The frequency of effect alleles is denoted by EAF, while β signifies the calculated genetic impact of exposure. A F statistic of 10 or more suggests a reduced risk of weak instrument bias within the MR analysis.We calculated the statistical power of the MR results using mRnd [34].

### Statistical analysis

**Fig. 1:**
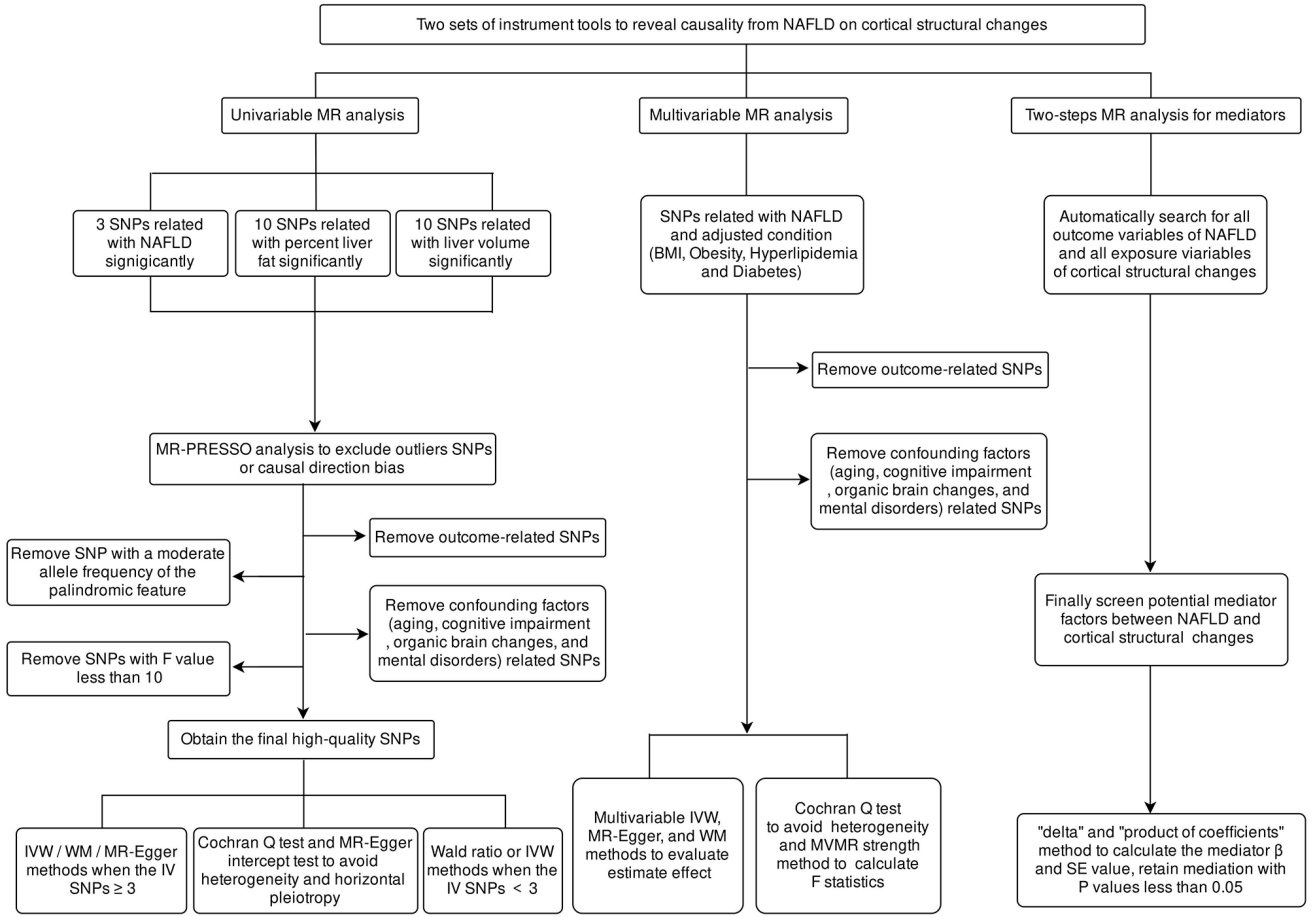
The flowchart of the Mendelian Randomization (MR) study. Based on mendelian assumption 1, NAFLD, liver fat and liver volume are significantly related to genetic instrumental variables (IVs). Assumption 2 proposes that these IVs do not influence outcomes via confounding variables. Assumption 3 asserts that the genetic IVs do not directly impact the structure of cortex but exert influence only through indirect exposure

The flowchart of the study is shown in Fig. 1. We utilized a two-sample MR approach which requires the exposure and outcome variables to satisfy three vital assumptions [35]: (i) there is a robust association between genetic variants and exposure, (ii) no correlation exists between genetic variants and any confounder associated with outcome variables, and (iii) genetic variants influence the outcome exclusively through exposure. We performed initial bidirectional two-sample MR analyses to examine causal associations of NAFLD, liver fat, liver volume with cortical SA and cortical TH. For under three IV SNPs, we employed either the Wald ratio or IVW method; for three or more SNPs, we utilized IVW, MR-Egger, and weighted median (WM) to improve result robustness and reduce heterogeneity or pleiotropy [32]. We primarily utilized IVW as the outcome measure, using MR-Egger and WM as supplementary techniques to strengthen result robustness across various conditions [35]. This approach accounted for pleiotropic effects and allowed for the use of potentially invalid instruments under specific circumstances [36, 37]. The IVW estimate was ascertained from the slope of the regression model regressing SNP-outcome on SNP-exposure effects. If inconsistent estimates were obtained, we refined the instrument *P*-value threshold and executed the MR analysis once more [38].

We conducted sensitivity analyses to gauge the validity and robustness of the results, which included employing the MR-Egger intercept test for the assessment of horizontal pleiotropy [36], and utilizing funnel plots to evaluate potential directional pleiotropy. We evaluated heterogeneity via Cochran’s Q test [39] and utilized MR-PRESSO to identify prospective outliers or directionality bias [31]. We implemented bidirectional MR and MR Steiger tests to preclude reverse causation and confirm directionality [40]. All above analyses were conducted using the “TwoSampleMR” and “MR-PRESSO” package. We used MRlap [41], a recent method correcting biases in Mendelian randomization analyses by addressing weak instrument bias and considering sample overlap as a modifier of these biases. MR Robust adjusted profile score (MR-RAPS) can provide a stable basis for inference in MR analysis through many weak instruments, especially with complex exposure-outcome relationships. We therefore conduct MR-RAPS as a supplementary method to verify the results of IVW [42, 43]. For global-level tests, a significant two-sided *P*-value was set at 0.05. For regional-level analyses, taking into account the 408 MR estimates, we applied the Bonferroni correction to set the *P*-value at 0.05/408 (1.22 × 10^− 4^).

We conducted multivariable MR analyses to ascertain whether the causal relationship between NAFLD and cortical SA or TH was independent of other NAFLD-associated factors including BMI, overweight status, hyperlipidemia, and diabetes [44]. We combined the genetic IVs from NAFLD and NAFLD-associated factors, additionally aggregating them through linkage disequilibrium (within a 1 MB window at R^2^ < 0.001) to guarantee SNP independence. Multivariable IVW, MR-Egger, WM and Lasso analyses were performed to evaluate estimate effect and correct for pleiotropy, with heterogeneity detected by Cochrane’s Q test, using the “MendelianRandomization” package [45, 46]. Total F-value of exposure variables was calculated with “MVMR” package [45, 46].

In a two-step MR analysis, we examined potential mediators demonstrating causal associations with both NAFLD and cortical structural alterations. Using the “GagnonMR” package, we implemented two-sample MR analyses covering all European ancestry variables (*n* = 18,115) in OPEN GWAS to identify putative mediators concurrently representing NAFLD outcome and cortical SA and TH exposure variables. We then employed the “product of coefficients” and “delta” method to assess the indirect influence of NAFLD on cortical SA and TH through each potential mediating variable. Mediators with a delta method *P*-value of less than 0.05 were ultimately considered significant and retained in our study [47, 48].

## Results

### Univariable MR

**Fig. 2:**
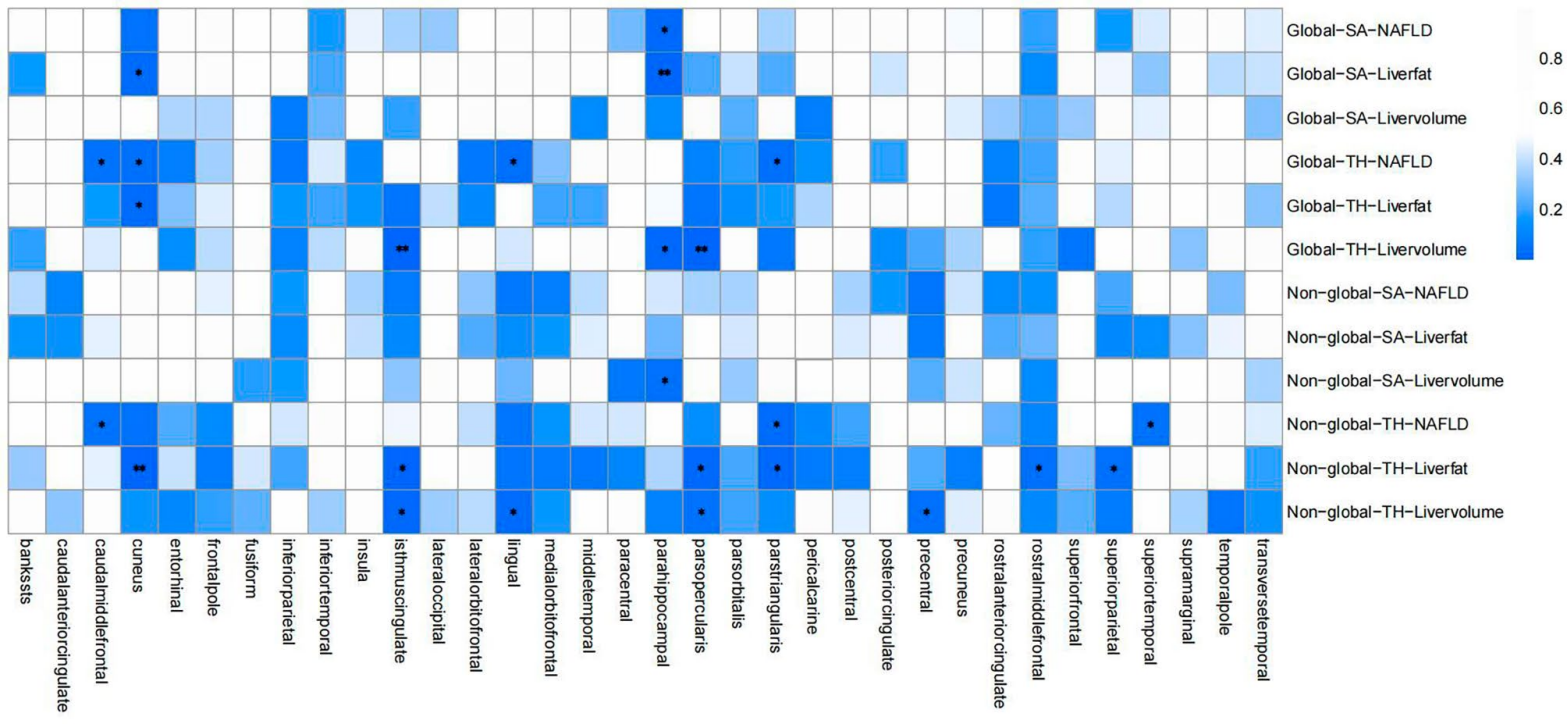
Heatmap of the inverse variance weighted (IVW) *P*-values from NAFLD, liver fat, and liver volume to cortical structural changes via MR analysis. The horizontal axis represents the corresponding brain regions being analyzed. The vertical axis indicates the exposure variables of interest - NAFLD, liver fat, and liver volume - along with the analytical conditions applied during processing, namely whether global weighting was employed and whether thickness or surface area of corresponding structural was examined. The color scale illustrates the *P*-values. Dark blue indicates lower *p*-values while white indicates higher *P*-values

Using Phenotype Scanner, we identified associations between particular SNPs (rs429358, rs9274447, rs4240624) and diseases including AD, schizophrenia, epilepsy, among others (Supplementary Table 3). After filtering these SNPs, we obtained 3, 10, and 10 IV SNPs respectively appropriate for the genetic prediction of NAFLD, liver fat, and liver volume, with no overlapping IV SNPs. MR-PRESSO showed no outliers or causal direction bias, and F statistics for all genetic IV SNPs exceeded 10, showing no weak instrument bias (Supplementary Table 2). We conducted a comprehensive MR study on overall SA/TH and 34 functional gyrus, incorporating NAFLD-related indicators. In global analyses, despite NAFLD, liver fat, and liver volume showing no significant causality with total cortical SA and TH, particular functional gyrus displayed nominally significant effects (Fig. 2). NAFLD significantly decreased parahippocampal SA without global weighting (b = -4.919 mm^2^, 95% CI: -9.024 to -0.814 mm, *P* = 0.019). In addition, the TH of the caudalmiddlefrontal (b = -0.005 mm, 95% CI: -0.010 to -0.0001 mm, *P* = 0.045), cuneus (b = -0.006 mm, 95% CI: -0.012 to -0.001 mm, *P* = 0.033), lingual (b = -0.005 mm, 95% CI: -0.010 to -0.0003 mm, *P* = 0.040), parstriangularis (b = -0.006 mm, 95% CI: -0.011 to -0.0002 mm, *P* = 0.040) was also effected. In non-global analyses, NAFLD decreased the caudalmiddlefrontal TH (b = -0.008 mm, 95% CI: -0.016 to -0.0002 mm, *P* = 0.044), parstriangularis TH (b = -0.009, 95% CI: -0.016 to-0.001 mm, *P* = 0.025), and superiotemporal SA (b = -21.607 mm^2^, 95% CI: -42.732 to -0.483 mm^2^, *P* = 0.045). Moreover, global weighting parahippocampal SA (b = -5.643 mm^2^, 95% CI:-9.658 to -1.629 mm^2^, *P* = 0.006) and non-global weighted parahippocampal SA (b = -9.851 mm^2^, 95% CI:-18.970 to -0.733 mm^2^, *P* = 0.034) were decreased by liver fat and volume respectively. Several other cortical regions, such as parsopercularis and isthmuscingulate, were also effected by these two exposure variables. Detailed information was shown in Table 1. Unfortunately, no results attained statistical significance after Bonferroni correction. The scatter plots demonstrate consistent trends in the observed causal effects for MR-Egger, WM, and IVW methods (Supplementary Fig. 1).

**Table 1:** Univariable MR results for the relationship between NAFLD, liver fat, liver volume and cortical structure

**NAFLD**									
	**Parahippocampal SA (global weighting)**	**Caudalmiddlefrontal TH (global weighting)**	**Cuneus TH (global weighting)**	**Lingual TH (global weighting)**	**Parstriangularis TH (global weighting)**	**Caudalmiddlefrontal TH (non-global weighting)**	**Parstriangularis TH (non-global weighting)**	**Superiotemporal SA (non-global weighting)**	
Inverse variance weighted	-4.919 (-9.024,-0.814) *P* = 0.019	-0.005 (-0.010,-0.0001) *P* = 0.045	-0.006 (-0.012,-0.001) *P* = 0.033	-0.005 (-0.010,-0.0003) *P* = 0.040	-0.006 (-0.011,-0.0002) *P* = 0.040	-0.008 (-0.016,-0.0002) *P* = 0.044	-0.009 (-0.016,-0.001) *P* = 0.025	-21.607 (-42.732,-0.483) *P* = 0.045	
**Liver fat**									
	**Cuneus SA (global weighting)**	**Parahippocampal SA (global weighting)**	**Parsopercularis TH (non-global weighting)**	**Cuneus TH (non-global weighting)**	**Isthmuscingulate TH (non-global weighting)**	**Parsopercularis TH (non-global weighting)**	**Parstriangularis TH (non-global weighting)**	**Rostralmiddlefrontal TH (non-global weighting)**	**Superiorparietal SA (non-global weighting)**
MR-Egger	12.130 (1.448,2.373E10) *P* = 0.090	-3.710 (-9.726,2.305) *P* = 0.266	-0.008 (-0.017,0.0002) *P* = 0.096	-0.008 (-0.018,0.002) *P* = 0.182	-0.0005 (-0.015,0.014) *P* = 0.950	-0.010 (-0.024,0.005) *P* = 0.239	-0.010 (-0.021,0.001) *P* = 0.131	-0.006 (-0.016,0.004) *P* = 0.267	-0.004 (-0.014,0.006) *P* = 0.418
Weighted median	10.695 (4.892,3.978E08) *P* = 0.021	-4.875 (-9.569,-0.182) *P* = 0.042	-0.007 (-0.014,-0.001) *P* = 0.032	-0.008 (-0.016,-0.001) *P* = 0.033	-0.010 (-0.021,0.002) *P* = 0.107	-0.011 (-0.022,0.0002) *P* = 0.054	-0.010 (-0.018,-0.001) *P* = 0.025	-0.007 (-0.015,0.001) *P* = 0.104	-0.006 (-0.014,0.002) *P* = 0.172
Inverse variance weighted	8.727 (2.191,1.735E07) *P* = 0.031	-5.643 (-9.658,-1.629) *P* = 0.006	-0.006 (-0.012,-0.001) *P* = 0.030	-0.009 (-0.016,-0.002) *P* = 0.008	-0.013 (-0.023,-0.003) *P* = 0.011	-0.012 (-0.022,-0.002) *P* = 0.014	-0.010 (-0.017,-0.002) *P* = 0.012	-0.007 (-0.014,-0.001) *P* = 0.034	-0.007 (-0.014,-0.001) *P* = 0.035
**Liver volume**									
	**Isthmuscingulate TH (global weighting)**	**Parahippocampal TH (global weighting)**	**Parsopercularis TH (global weighting)**	**Isthmuscingulate TH (non-global weighting)**	**Lingual TH (non-global weighting)**	**Parahippocampal SA (non-global weighting)**	**Parsopercularis TH (non-global weighting)**	**Precentral TH (non-global weighting)**	
MR-Egger	-0.028 (-0.065,0.009) *P* = 0.179	0.023 (-0.034,0.081) *P* = 0.453	-0.017 (-0.036,0.002) *P* = 0.123	-0.040 (-0.083,0.003) *P* = 0.130	-0.011 (-0.035,0.013) *P* = 0.401	-3.809 (-24.251,16.632) *P* = 0.726	-0.020 (-0.048,0.009) *P* = 0.229	-0.007 (-0.038,0.024) *P* = 0.679	
Weighted median	-0.027 (-0.05,-0.004) *P* = 0.021	0.023 (-0.011,0.057) *P* = 0.187	-0.014 (-0.025,-0.002) *P* = 0.024	-0.025 (-0.051,0.001) *P* = 0.061	-0.012 (-0.027,0.004) *P* = 0.135	-9.246 (-21.528,3.036) *P* = 0.140	-0.012 (-0.031,0.006) *P* = 0.202	-0.014 (-0.032,0.005) *P* = 0.144	
Inverse variance weighted	-0.027 (-0.044,-0.01) *P* = 0.002	0.033 (0.006,0.059) *P* = 0.017	-0.012 (-0.021,-0.004) *P* = 0.006	-0.026 (-0.047,-0.005) *P* = 0.015	-0.013 (-0.024,-0.001) *P* = 0.030	-9.851 (-18.97,-0.733) *P* = 0.034	-0.015 (-0.03,-0.001) *P* = 0.043	-0.016 (-0.03,-0.001) *P* = 0.037	

NAFLD, Non-Alcoholic Fatty Liver Disease; MR, mendelian randomization; SA, surface area; TH, thickness

Although our MR analysis did not reveal significant evidence of horizontal pleiotropy or heterogeneity (Supplementary Table 4), we did observe slight asymmetry in certain funnel plots (Supplementary Fig. 1). Additionally, we did not detect any inproper directionality of the causal relationship in the MR Steiger test analysis (Supplementary Table 5). However, bidirectional MR results suggested potential associations between non-global weighing isthmuscingulate TH and cuneus TH and liver fat, and between non-global parahippocampal SA and liver volume (Supplementary Table 6).

With MRlap correction, associations of NAFLD with decreased cortical TH and SA in the Caudalmiddlefrontal, Cuneus, Parstriangularis, and Superiotemporal regions were no longer significant (*P* > 0.05), potentially due to the limited number of IV SNPs. However, negative correlations persisted across these regions (Supplementary Table 7). Critically, links between liver fat/volume and cortical structure remained significant, affirming that sample overlap likely does not substantially impact conclusions. Though some instruments displayed high F-statistics yet low statistical power (Supplementary Table 2), MR-RAPS results aligned with inverse variance weighted analysis (Supplementary Table 8).

### Multivariable MR

Supplementary Table 1 shows the sources and details of the GWAS information employed in the multivariable MR analyses. The independent IV SNPs for multivariable MR are documented in Supplementary Table 9. After adjusting for BMI, diabetes, hyperlipidemia, and obesity, NAFLD continued exhibiting significant impacts on global-weighted lingual TH and non-global weighted parstriangularis TH, with the corresponding regression coefficients and confidence interval as follows: BMI (b = -0.004 mm, 95% CI: -0.008 to -0.001 mm, *P* = 0.013 & b = -0.007 mm, 95% CI: -0.012 to -0.001 mm, *P* = 0.018), Diabetes (b = -0.006 mm, 95% CI: -0.011 to -0.001 mm, *P* = 0.012 & b = -0.007 mm, 95% CI: -0.015 to 0.000 mm, *P* = 0.047), Hyperlipidemia (b = -0.006 mm, 95% CI: -0.012 to -0.001 mm, *P* = 0.014 & b = -0.009 mm, 95% CI: -0.017 to -0.001 mm, *P* = 0.022), and Obesity (b = -0.006 mm, 95% CI: -0.011 to -0.002 mm, *P* = 0.008 & b = -0.007 mm, 95% CI: -0.015 to -0.000 mm, *P* = 0.045). The confidence interval derived from the multivariable MR-Egger and WM were broader as compared to those from the multivariable IVW, which provided more robust estimates in a broader context. Furthermore, Under certain conditions, NAFLD might exert an independent causal effect on other cortical structures. For example, after adjusting for BMI, the impact of NAFLD on cuneus TH, lingual TH, caudal middle frontal TH, and parstriangularis TH remained significant. Similar results were observed after other factor adjustments. The LASSO regression results were almost consistent with those of the MVMR-IVW analyses. Detailed information can be found in Table 2. The MVMR-RAPS results demonstrate that NAFLD exhibits independent effect on the previously identified cortical structural alterations from two-sample MR analyses, after adjusting for BMI, diabetes, hyperlipidemia and obesity (Supplementary Table 10). This could be explained by MR-RAPS being less subject to conditionally weak instrument bias.

**Table 2:** Multivariable MR results for the relationship between NAFLD and cortical structure, adjusted for BMI, diabetes, hyperlipidemia, and obesity

	Parahippocampal SA (global weighting)	Caudalmiddlefrontal TH (global weighting)	Cuneus TH (global weighting)	lingual TH (global weighting)	Parstriangularis TH (global weighting)	Caudalmiddlefrontal TH (non-global weighting)	Parstriangularis TH (non-global weighting)	Superiotemporal SA (non-global weighting)
Adjusted for BMI								
MR-Egger	-2.818 (-6.680, 1.044) *P* = 0.153	-0.004 (-0.009, 0.000) *P* = 0.055	-0.004 (-0.009, 0.001) *P* = 0.149	-0.003 (-0.007, 0.001) *P* = 0.186	-0.002 (-0.007, 0.003) *P* = 0.496	-0.007 (-0.014, -0.001) *P* = 0.033	-0.006 (-0.013, 0.001) *P* = 0.076	-5.006 (-28.137, 18.126) *P* = 0.671
Weighted median	-4.193 (-8.033, -0.354) *P* = 0.032	0.002 (-0.003, 0.008) *P* = 0.354	-0.006 (-0.011, -0.001) *P* = 0.024	-0.006 (-0.010, -0.001) *P* = 0.016	-0.002 (-0.008, 0.003) *P* = 0.345	0.000 (-0.007, 0.007) *P* = 1.000	-0.008 (-0.014, -0.001) *P* = 0.025	-17.636 (-37.431, 2.159) *P* = 0.081
Inverse variance weighted	-2.662 (-5.720, 0.396) *P* = 0.088	0.000 (-0.004, 0.004) *P* = 0.967	-0.005 (-0.009, -0.001) *P* = 0.011	-0.004 (-0.008, -0.001) *P* = 0.013	-0.001 (-0.005, 0.003) *P* = 0.649	-0.005 (-0.011, 0.000) *P* = 0.049	-0.007 (-0.012, -0.001) *P* = 0.018	-15.500 (-33.914, 2.915) *P* = 0.099
Lasso	-3.362 (-6.107, -0.616) *P* = 0.016	0.000 (-0.003, 0.004) *P* = 0.970	-0.007 (-0.011, -0.003) *P* = 0.001	-0.005 (-0.008, -0.001) *P* = 0.008	-0.001 (-0.005, 0.002) *P* = 0.537	-0.005 (-0.010, 0.000) *P* = 0.050	-0.007 (-0.012, -0.002) *P* = 0.005	-18.391 (-32.895, -3.887) *P* = 0.013
BMI F statistics	48.515	48.515	48.515	48.515	48.515	48.515	48.515	48.515
Adjusted for Diabetes								
MR-Egger	-3.642 (-8.142, 0.859) *P* = 0.113	-0.005 (-0.010, 0.001) *P* = 0.088	-0.006 (-0.012, 0.000) *P* = 0.052	-0.008 (-0.013, -0.002) *P* = 0.009	-0.005 (-0.012, 0.002) *P* = 0.164	-0.007 (-0.015, 0.001) *P* = 0.081	-0.009 (-0.017, 0.000) *P* = 0.048	-29.939 (-54.843, -5.035) *P* = 0.018
Weighted median	-3.989 (-8.633, 0.654) *P* = 0.092	-0.004 (-0.010, 0.003) *P* = 0.245	-0.006 (-0.012, 0.001) *P* = 0.090	-0.006 (-0.012, 0.000) *P* = 0.040	-0.005 (-0.011, 0.001) *P* = 0.113	-0.007 (-0.016, 0.001) *P* = 0.102	-0.008 (-0.017, 0.000) *P* = 0.064	-18.695 (-42.590, 5.200) *P* = 0.125
Inverse variance weighted	-4.432 (-8.251, -0.612) *P* = 0.023	-0.003 (-0.008, 0.001) *P* = 0.144	-0.005 (-0.010, 0.000) *P* = 0.067	-0.006 (-0.011, -0.001) *P* = 0.012	-0.003 (-0.009, 0.003) *P* = 0.282	-0.007 (-0.014, -0.001) *P* = 0.035	-0.007 (-0.015, 0.000) *P* = 0.047	-19.652 (-41.725, 2.420) *P* = 0.081
Lasso	-4.432 (-8.251, -0.612) *P* = 0.023	-0.003 (-0.008, 0.001) *P* = 0.144	-0.005 (-0.010, 0.000) *P* = 0.067	-0.006 (-0.011, -0.002) *P* = 0.008	-0.004 (-0.009, 0.000) *P* = 0.068	-0.007 (-0.014, -0.001) *P* = 0.035	-0.007 (-0.015, 0.000) *P* = 0.047	-21.574 (-40.413, -2.735) *P* = 0.025
DiabetesF statistics	43.204	43.204	43.204	43.204	43.204	43.204	43.204	43.204
Adjusted for Hyperlipidemia								
MR-Egger	-2.533 (-8.001, 2.934) *P* = 0.364	-0.007 (-0.014, 0.000) *P* = 0.047	-0.007 (-0.017, 0.004) *P* = 0.200	-0.006 (-0.013, 0.000) *P* = 0.066	-0.010 (-0.019, -0.001) *P* = 0.024	-0.009 (-0.020, 0.001) *P* = 0.075	-0.014 (-0.024, -0.003) *P* = 0.008	-29.587 (-57.806, -1.369) *P* = 0.040
Weighted median	-4.214 (-9.651, 1.224) *P* = 0.129	-0.006 (-0.012, 0.001) *P* = 0.106	-0.004 (-0.012, 0.003) *P* = 0.263	-0.006 (-0.013, 0.000) *P* = 0.062	-0.005 (-0.012, 0.002) *P* = 0.135	-0.006 (-0.016, 0.004) *P* = 0.217	-0.007 (-0.017, 0.004) *P* = 0.216	-9.672 (-38.804, 19.460) *P* = 0.515
Inverse variance weighted	-4.199 (-8.319, -0.079) *P* = 0.046	-0.005 (-0.010, 0.001) *P* = 0.081	-0.004 (-0.012, 0.003) *P* = 0.255	-0.006 (-0.012, -0.001) *P* = 0.014	-0.007 (-0.013, 0.000) *P* = 0.054	-0.006 (-0.013, 0.002) *P* = 0.149	-0.009 (-0.017, -0.001) *P* = 0.022	-12.533 (-35.959, 10.893) *P* = 0.294
Lasso	-4.199 (-8.319, -0.079) *P* = 0.046	-0.005 (-0.010, 0.001) *P* = 0.081	-0.004 (-0.012, 0.003) *P* = 0.255	-0.006 (-0.012, -0.001) *P* = 0.014	-0.007 (-0.013, 0.000) *P* = 0.054	-0.006 (-0.013, 0.002) *P* = 0.149	-0.009 (-0.017, -0.001) *P* = 0.022	-12.533 (-35.959, 10.893) *P* = 0.294
Hyperlipidemia F statistics	18.654	18.654	18.654	18.654	18.654	18.654	18.654	18.654
Adjusted for Obesity								
MR-Egger	-5.900 (-11.114, -0.686) *P* = 0.027	-0.008 (-0.016, -0.001) *P* = 0.031	-0.003 (-0.015, 0.008) *P* = 0.575	-0.005 (-0.011, 0.002) *P* = 0.170	-0.011 (-0.018, -0.005) *P* = 0.001	-0.009 (-0.025, 0.007) *P* = 0.267	-0.012 (-0.022, -0.003) *P* = 0.012	-27.064 (-63.955, 9.826) *P* = 0.150
Weighted median	-4.172 (-8.817, 0.473) *P* = 0.078	-0.005 (-0.011, 0.001) *P* = 0.119	-0.006 (-0.013, 0.001) *P* = 0.078	-0.006 (-0.012, 0.000) *P* = 0.049	-0.005 (-0.011, 0.001) *P* = 0.129	-0.007 (-0.016, 0.002) *P* = 0.117	-0.008 (-0.016, 0.001) *P* = 0.074	-17.546 (-41.649, 6.557) *P* = 0.154
Inverse variance weighted	-4.417 (-8.138, -0.696) *P* = 0.020	-0.003 (-0.010, 0.003) *P* = 0.339	-0.006 (-0.015, 0.002) *P* = 0.117	-0.006 (-0.011, -0.002) *P* = 0.008	-0.003 (-0.011, 0.004) *P* = 0.397	-0.006 (-0.017, 0.004) *P* = 0.230	-0.007 (-0.015, 0.000) *P* = 0.045	-15.750 (-41.573, 10.072) *P* = 0.232
Lasso	-4.417 (-8.138, -0.696) *P* = 0.020	-0.003 (-0.010, 0.003) *P* = 0.339	-0.006 (-0.011, -0.001) *P* = 0.026	-0.006 (-0.011, -0.002) *P* = 0.008	-0.005 (-0.010, 0.000) *P* = 0.038	-0.006 (-0.013, 0.001) *P* = 0.083	-0.009 (-0.016, -0.002) *P* = 0.009	-15.750 (-41.573, 10.072) *P* = 0.232
Obesity F statistics	41.334	41.334	41.334	41.334	41.334	41.334	41.334	41.334

NAFLD, Non-Alcoholic Fatty Liver Disease; MR, mendelian randomization; SE, standard error; CI, confidential interval; SA, surface area; TH, thickness

### Two-steps MR

**Fig. 3:**
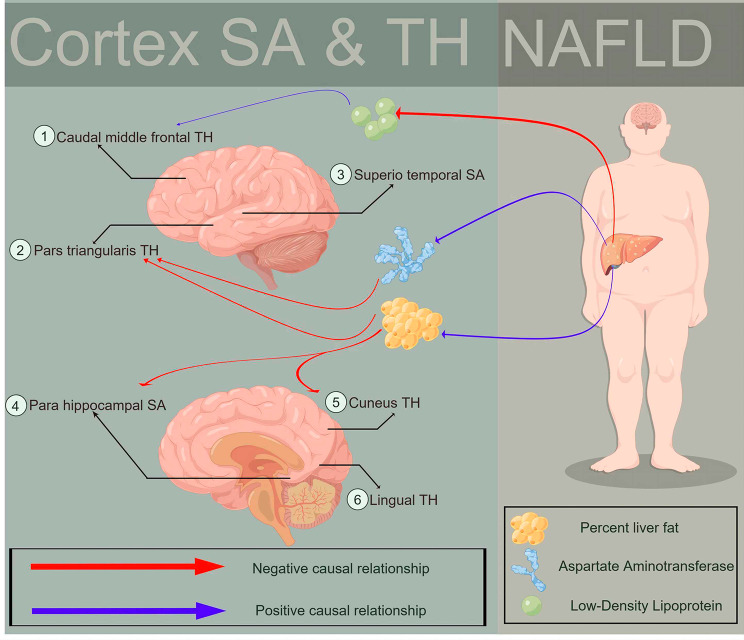
The potential mediator mechanism (percent liver fat, aspartate aminotransferase, low-density lipoprotein) of cortical structure changes from NAFLD via MR analysis

In two-step MR analyses, we investigated potential mediators of the causal associations between NAFLD and cortical structure. In the first step, we utilized genetic instruments for NAFLD to evaluate its causal effects on prospective mediators, and screened 1,368 potential outcomes among 18,115 European ancestry variables. In the second step, with above-mentioned influenced cortex as the outcome variables, we screened 139, 244, 246, 175, 200, 297, 214, 272 potential exposure factors, respectively. We took the intersection and excluded variables of: i) drug use; ii) body characteristics; iii) common blood test indices; iv) lipid micromorphology. Priority was given to the most updated version or deeper sequenced version. Supplementary Table 11 presents the sources and details of GWAS data for the potential mediators after preliminary screening. Employing the ‘product of coefficients’ and the ‘delta’ methods, we discerned a significant mediating role of liver fat percentage in the causal association between NAFLD and global weighting parahippocampal SA (IVW β = -4.766; 95% CI, -7.836 to -1.697; *P* = 0.002), global weighting cuneus TH (IVW β = -0.006; 95% CI: -0.010 to -0.002; *P* = 0.07), and non-global weighting parstriangularis TH (IVW β = -0.007; 95% CI: -0.013 to -0.001; *P* = 0.017); AST had a significant mediating effect in the causal association from NAFLD to non-global weighting parstriangularis TH (IVW β = -0.007; 95% CI: -0.011 to -0.002; *P* = 0.002); LDL had a significant mediating effect in the causal association from NAFLD to non-global weighting caudalmiddlefrontal TH (IVW β = -0.0003; 95% CI: -5.650E-04 to -1.390E-05; *P* = 0.040) (Fig. 3; Table 3). All the mediation effect of potential mediators were shown in Supplementary Table 12.

**Table 3:** The mediation effect of NAFLD on cortical construction via liver fat, LDL cholesterol and AST

Exposure-Outcome	Mediator	Total effect Beta (95% CI)	Direct effect A Beta (95% CI)	Direct effect B Beta (95% CI)	Mediation effect Beta (95% CI)	Mediated *P* value
NAFLD-Parahippocampal SA (global weighting )	Percent liver fat	-4.919 (-9.023, -0.814)	0.786 (0.722, 0.850)	-6.064 (-9.939, -2.190)	-4.766 (-7.836, -1.697)	0.002
NAFLD-Cuneus TH (global weighting )	Percent liver fat	-0.006 (-0.012, -0.001)	0.786 (0.722, 0.850)	-0.008 (-0.013, -0.002)	-0.006 (-0.010, -0.002)	0.007
NAFLD-Caudalmiddlefrontal TH (non-global weighting )	LDL cholesterol	-0.008 (-0.016, -0.001)	-0.026 (-0.043, -0.009)	0.0111 (0.004, 0.019)	-0.0003 (-5.650E-04, -1.390E-05)	0.040
NAFLD-Parstriangularis TH (non-global weighting )	Percent liver fat	-0.009 (-0.016, -0.001)	0.786 (0.722, 0.850)	-0.009 (-0.016, -0.002)	-0.007 (-0.013, -0.001)	0.017
NAFLD-Parstriangularis TH (non-global weighting )	AST	-0.009 (-0.016, -0.001)	3.529 (3.321, 3.737)	-0.002 (-0.003, -0.001)	-0.007 (-0.011, -0.002)	0.002

NAFLD: Non-Alcoholic Fatty Liver Disease; LDL: Low-Density lipoprotein; AST: Aspartate aminotransferase; SA, surface area; TH, thickness

## Discussion

Accumulated evidence indicates an association between NAFLD and neuro-pathologies, impacting both cognitive function and brain volume. Animal studies have demonstrated NAFLD effects on thalamic energy metabolism and hippocampal structure [6, 9, 49]. We used genetic tools for liver fat and liver volume to represent the liver health status in NAFLD [28, 29]. In MRI results, SA and TH served as indicators of cortical structure and alterations in neurological function. This study is the first large-scale MR analysis to conclusively establish a causal link between NAFLD, liver fat, liver volume, and cortical structure. The association was established by using genetic variations as unbiased proxies, an approach not extensively used in previous cross-sectional or experimental studies [7, 21, 49].

Cognitive and neuropsychiatric disturbances demonstrate higher prevalence among NAFLD patients, however the distinct cortical substrates and pathogenesis have not been fully elucidated [50, 51]. Specific attributes of brain structure, including cortical SA and TH, might serve as predictors for cognitive decline. While older theories linked brain damage from liver disease to factors like ammonia toxicity and neurotransmitter changes [52], the more recent liver-brain axis hypothesis suggests direct communication pathways between liver disease and brain injury. These pathways may involve liver-derived substances modifying CNS proteins through nitrotyrosine or nitrosamine, ammonia accumulation triggering phagocytosis in brain glial cells, and systemic inflammation affecting the integrity of the blood-brain barrier [53–55]. In our MR analysis, we examined the heterogeneity and severity of NAFLD, revealing its association with structural brain changes, including potential alterations to the cortical SA and TH in regions like the parahippocampus. Despite statistical insignificance per Bonferroni-corrected thresholds, associations surpassing *P* < 0.05 in IVW assessment deserve prudent examination. In concert with extant evidence, our research underscore the import of cortical integrity to the pathology underlying the hepatic-cerebral connection.

In our exposure instrumental variables, rs28601761, rs73001065, and rs3747207 are three IV SNPs closely associated with NAFLD. Positioned downstream of the TRIB1 gene, rs28601761 is significantly correlated with serum levels of glycine and tyrosine, suggesting its potential involvement in NAFLD by influencing lipid metabolism [56]. Located in the MAU2 gene, rs73001065 exhibits different genotypes associated with serum total cholesterol, low-density lipoprotein cholesterol, apolipoprotein B, and triglyceride levels. It may contribute to gene-gene and gene-environment interactions, influencing lipid levels and regulating the progression of NAFLD [57]. Positioned within the PNPLA3 gene, rs3747207, although unrelated to liver cancer susceptibility, is significantly correlated with the severity and activity score (NAS) of NAFLD. This SNP represents the strongest genetic signal associated with NAFLD in the PNPLA3 gene region [58].

Our study identified a significant negative impact of NAFLD, liver fat, and liver volume on the structure of the parahippocampus, a crucial component of the cortico-limbic subcortical loop that plays a role in emotional behavior and memory processing. Reductions in hippocampus and parahippocampal gyrus volumes are observed in psychiatric conditions such as depression, schizophrenia, and AD [59–61]. Additionally, abnormalities in parahippocampal gyrus activity were noted in cirrhosis and hepatic HE patients [62, 63], possibly related to glucose metabolism changes [64]. Moreover, Hepatitis C virus (HCV) patients showed noticeable grey matter atrophy in the left parahippocampal gyrus [65], while better liver function correlates with larger parahippocampal volume [66]. Consistent with previous evidence of superiortemporal gyrus atrophy in minimal hepatic encephalopathy (MHE) [67], our findings revealed a negative correlation between superiortemporal surface area and NAFLD risk. Glucose metabolism decreased in HCV patients’ superiortemporal region [64]. In cirrhosis patients, superiortemporal region fALFF significantly increased 6 months post-TIPS surgery compared to 3 months [68].

Cuneus and precuneus, crucial to visual and cognitive processing. Irregularities in cortical brain regions, including the cuneus and precuneus, persisted in patients with cirrhosis and HE, even after undergoing liver transplantation [69]. Meanwhile, patients with cirrhosis and HE also demonstrate notable reductions in fALFF in regions such as the precuneus and cerebellum posterior lobe [62]. Of note, we observed that alterations in isthmuscingulate and cuneus cortical thickness appeared to conversely influence liver fat percentage, potentially attributable to CNS-liver bidirectional signaling governing hepatic lipid and lipoprotein regulation [70]. The relation between NAFLD and the SA of the caudalmiddlefrontal gyrus is yet to be established. Sun et al. reported heightened functional connectivity in patients with cirrhosis, potentially linked to liver dysfunction [71]. Cirrhosis patients were found to have cerebral blood flow deficiencies in the middlesuperiorfrontal and inferiorparietal lobe [72], with neuropsychological performance and ammonia levels linked to changes in the right middlefrontal gyrus [70].

The parstriangularis and parsopercularis have been found associated with NAFLD and liver fat/volume, respectively. These brain regions constitute the inferior frontal gyrus, which plays critical roles in response inhibition, motor inhibition, and social cognition [73]. Multiple studies have observed functional abnormalities in the inferior frontal gyrus among HE patients, as well as reduced functional connectivity between right dorsolateral prefrontal cortex and inferior frontal gyrus among cirrhotic patients [55, 74]. Additionally, superiorparietal and precentral structures have shown vulnerability to effects of liver fat and volume. Perfusion of the former could be improved by branched-chain amino acid solutions in cirrhotic patients [75]. Smaller volumes in the latter have been associated with neurolesions related to alcohol use disorders (AUD) [76]. Certain findings appeared contradictory, including the observed positive correlations between liver fat and cuneus TH, as well as between liver volume and parahippocampus SA. These relationships, absent of detected pleiotropy, may potentially be explained by compensatory cortical hypertrophy or cerebral edema induced by liver conditions [77].

Our two-step MR analyses implied liver fat may mediate parahippocampal, cuneus, and parstriangularis structural alterations induced by NAFLD. NAFLD could also affect parstriangularis and caudalmiddlefrontal cortical thickness through changes in AST and LDL levels, respectively. Studies in German and Netherlands obese middle-aged/elderly cohorts evince liver fat-brain structure links independent of potential confounders [78, 79]. Elevated liver fat was found to correlate with lower total brain and gray matter blood flow [80], suggesting independent mechanisms affecting brain circulation and structure. Regarding AST, its serum disorders were linked with poorer clinical outcomes in stroke patients [81], and an aging-related study showed aging and oxidase may affect the brain starting from the liver [82]. Augmented AST and ALT manifest in depressed Korean females, implicating AST in neurological comorbidities [83]. Although LDL is considered risky for cardiovascular disease and atherosclerosis, recent research indicated its potential protective role in neuroinflammation [84]. A Chinese study found low LDL levels might associate with microstructural brain damage [85], and a higher LDL/HDL ratio could impact T2DM patients’ cognitive function by damaging functional connectivity [86]. Circulating ammonia, HDL or additional hepatic biomarkers failed to demonstrate significant mediation between NAFLD and cortical outcomes, potentially attributable to our “product of coefficients” and “delta” two-step approach. Accordingly, adequately-powered clinical observational and preclinical analyses remain imperative for clarifying precise pathways interconnecting NAFLD and cortical pathology.

### Study strengths and limitations

Our bidirectional two-sample MR study makes full use of extensive GWAS meta-analysis data and applies rigorous, detailed statistical methodologies. Primarily focused on individuals of European descent, the study examines the link between genetic susceptibility to NAFLD and changes in cortical MRI, including potential mediating mechanisms. Moreover, we applied analytical strategies, like bidirectional MR and MR Steiger tests, which enabled causality determination and limited potential confounding influences. However, it has limitations. Firstly, the SNPs only account for a fraction of the genetic variation related to NAFLD, with the biological role of these genetic markers remaining unclear. This uncertainty may challenge assumptions of independence and exclusion restrictions, notably regarding pleiotropy. Secondly, participation biases could persist within UK Biobank subgroups for hepatic and neuroimaging data. However, prior study demonstrate stronger IVs in two-sample MR analysis mitigate sample overlap bias [87]. We also attempted to minimize such bias via MRlap method. Thirdly, the variables in MR analysis reflects lifelong exposure, not short-term intervention. NAFLD and liver fat, despite their genetic correlations, can be reversed and are influenced by short-term lifestyle changes, necessitating prospective cohort studies. Fourthly, environmental/social elements may introduce bias in unrelated participants, exacerbated by multi-site GWAS provenance, warranting familial GWAS in subsequent analyses.

## Conclusion

our comprehensive MR analysis is the first to establish a causal link between NAFLD and cortical structure changes, particularly in parahippocampal, cuneus, and other cortical regions. Conducting brain MRI exams for patients with NAFLD may assist in the early diagnosis of psychiatric disorders. When changes in the isthmuscingulate and cuneus are observed, neurologists should also pay attention to the patient’s liver function. The specific link between NAFLD and cortical structure warrants further research.

### Electronic supplementary material

Below is the link to the electronic supplementary material.

Supplementary Material 1

Supplementary Material 2

## Data Availability

The datasets used and/or analyzed during the current study are available from the corresponding author on reasonable request.
