# Supplementary material for: Bidirectional two-sample mendelian randomization analysis identifies causal associations of MRI-based cortical thickness and surface area relation to NAFLD
Source: Lipids Health Dis. 2024 Feb 23;23:58. doi: 10.1186/s12944-024-02043-x (PMC10885469; doi:10.1186/s12944-024-02043-x)
Supplement: Supplementary file 2 — Supplementary Material 2 [file 12944_2024_2043_MOESM2_ESM.docx]

**
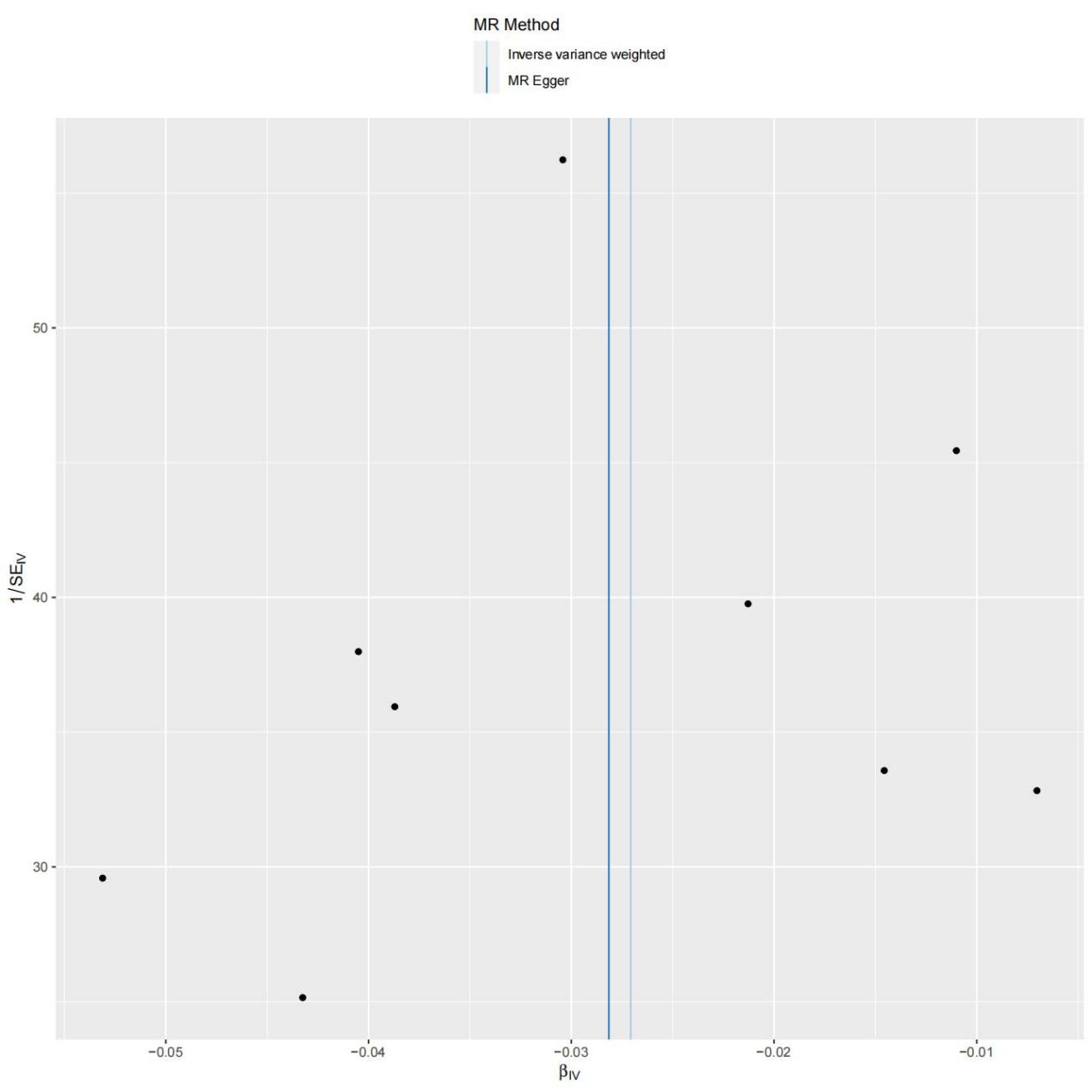

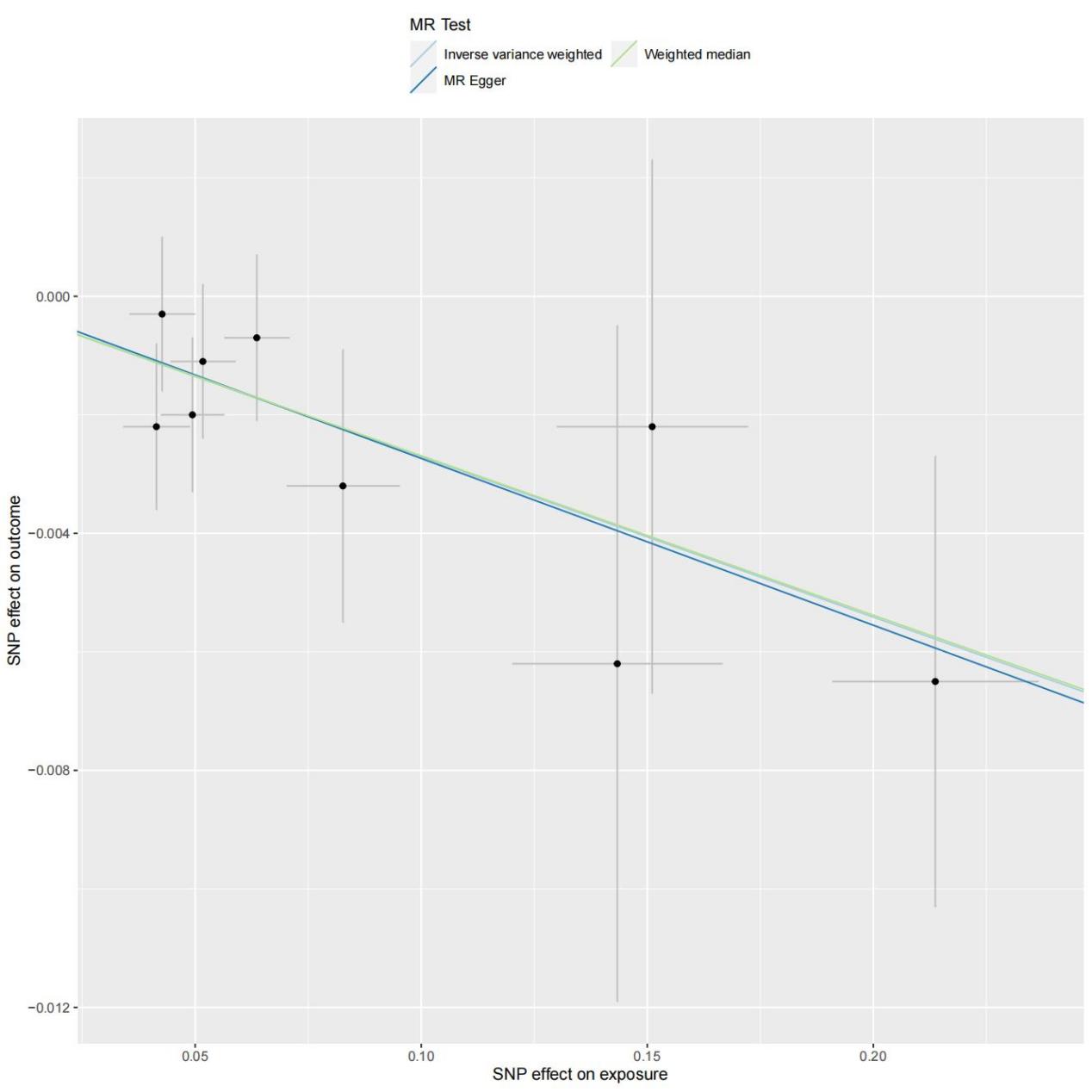
**

**a
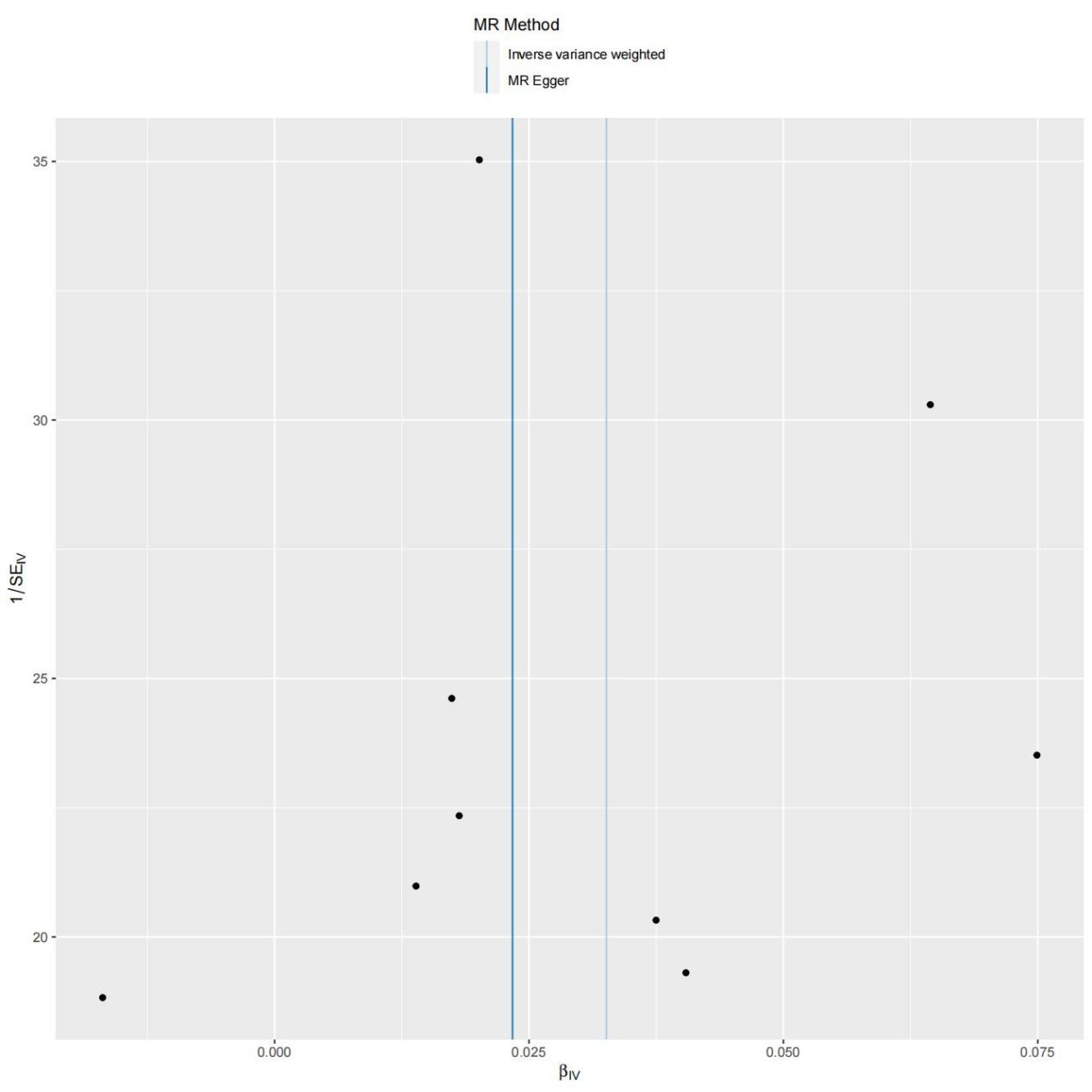

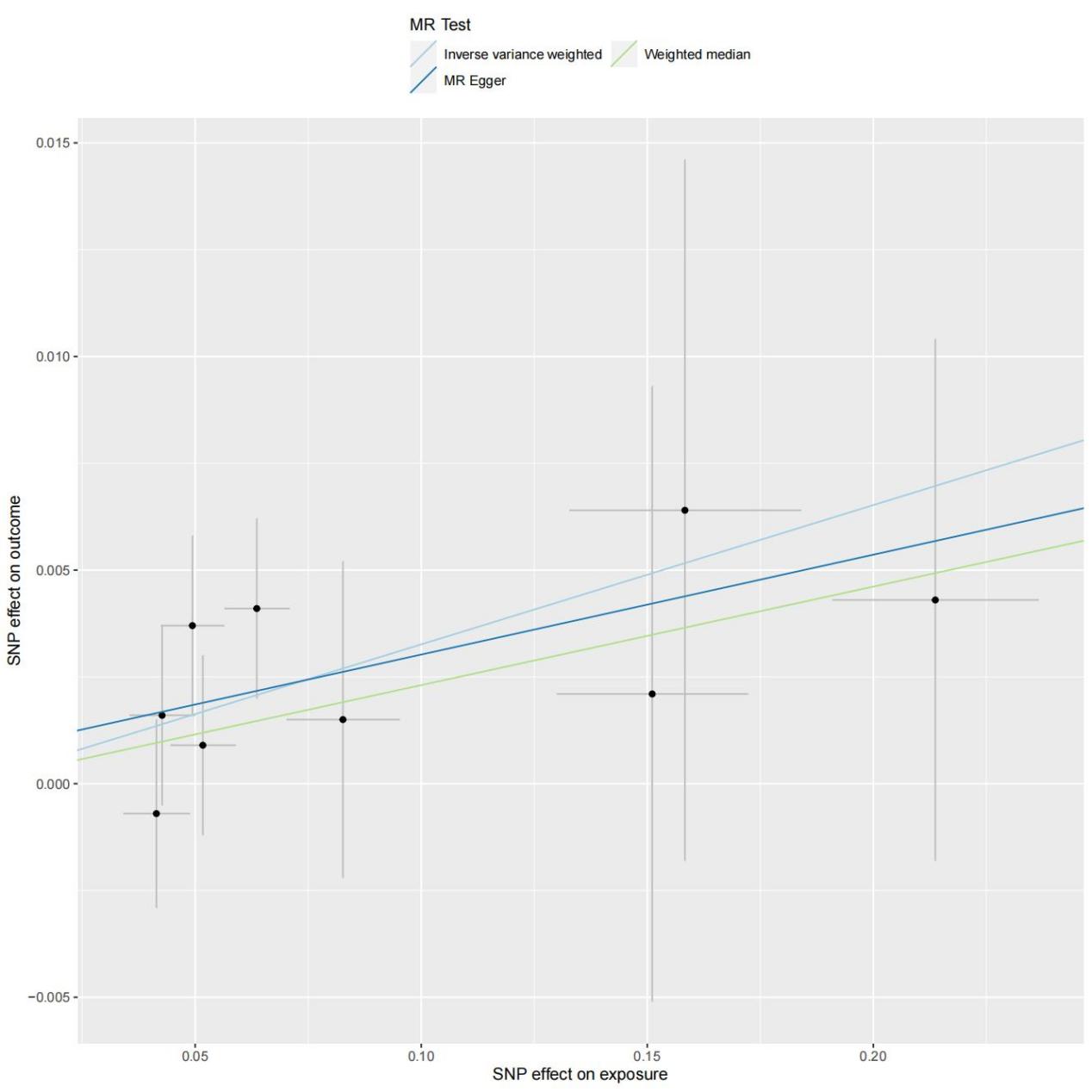
**

**b**

**
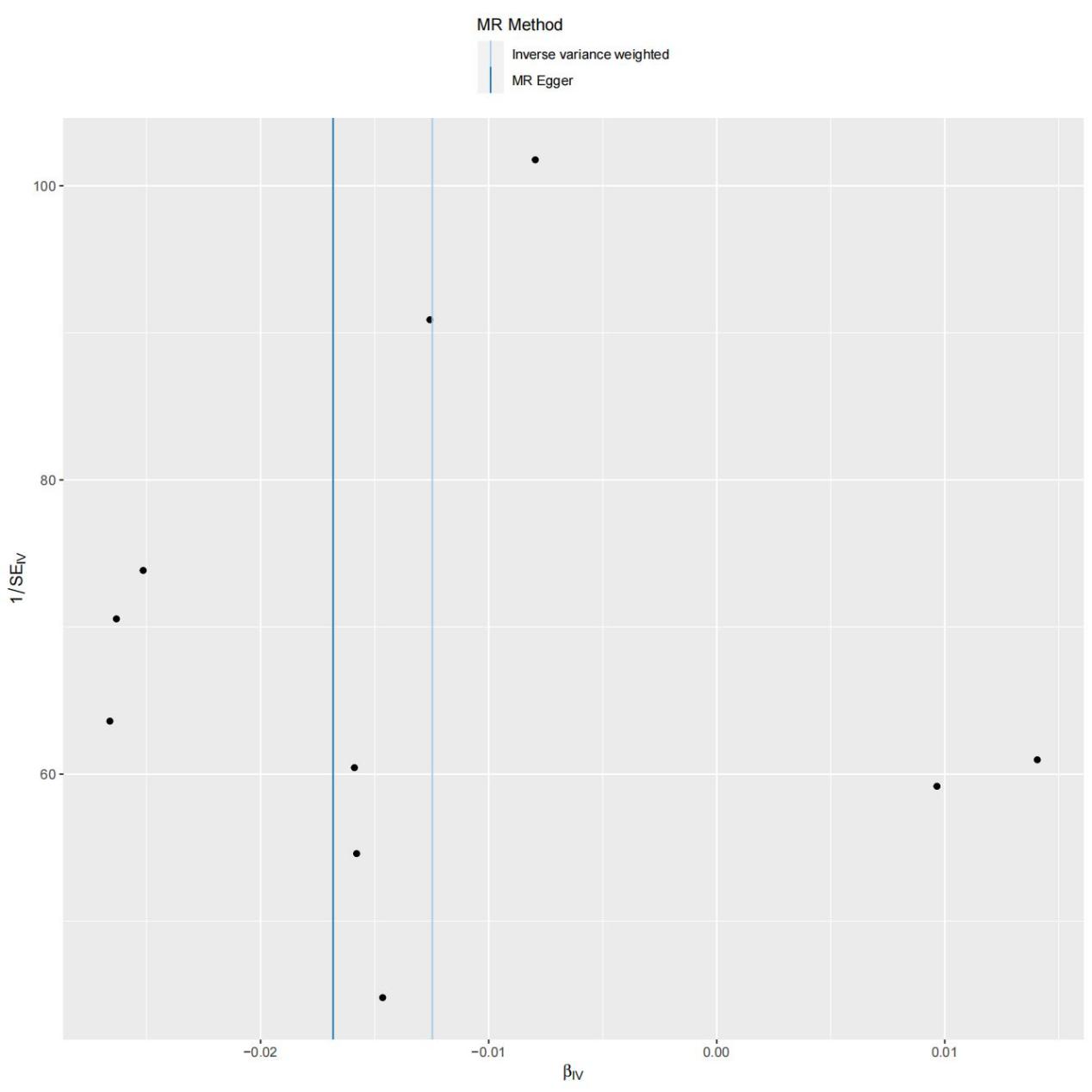

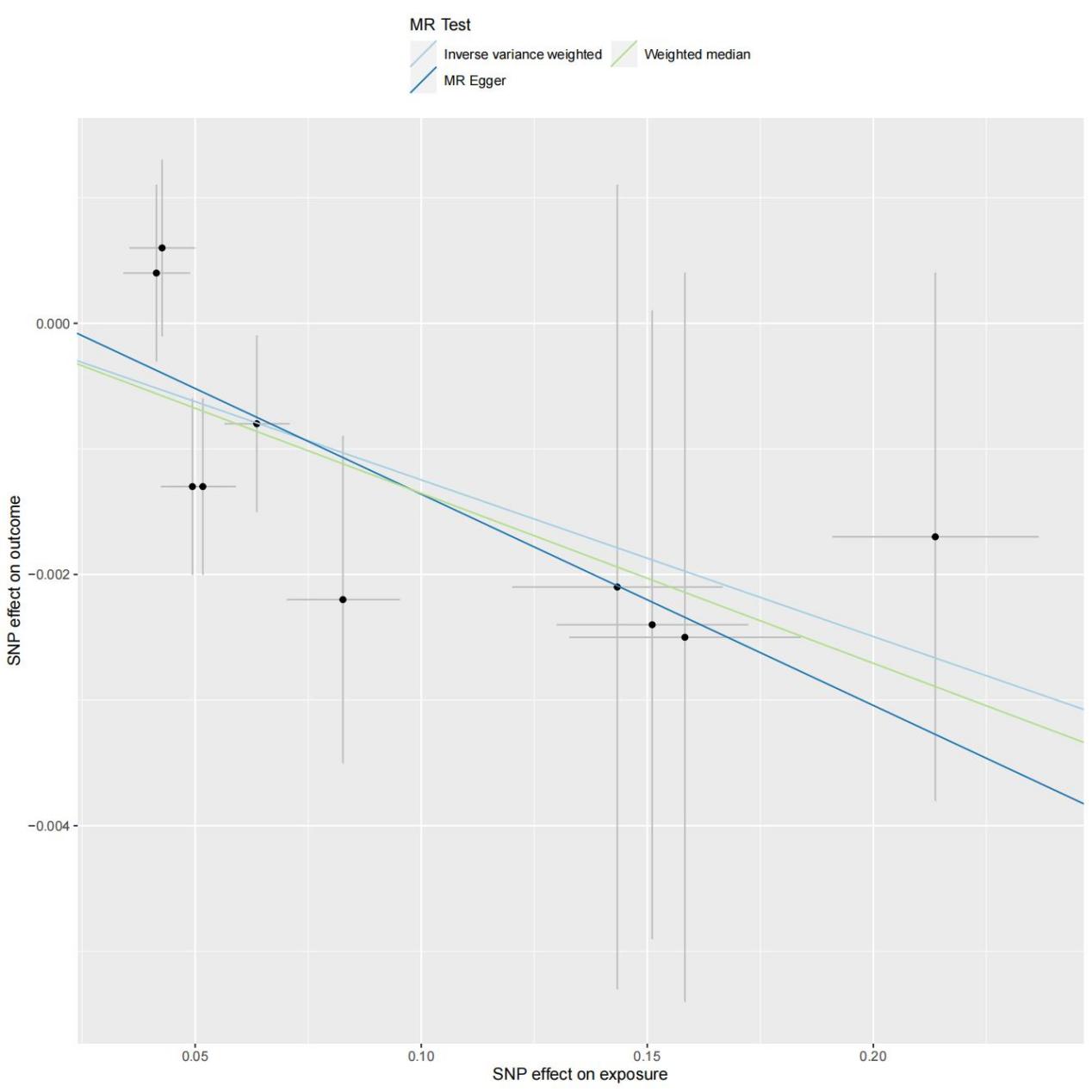
**

**c
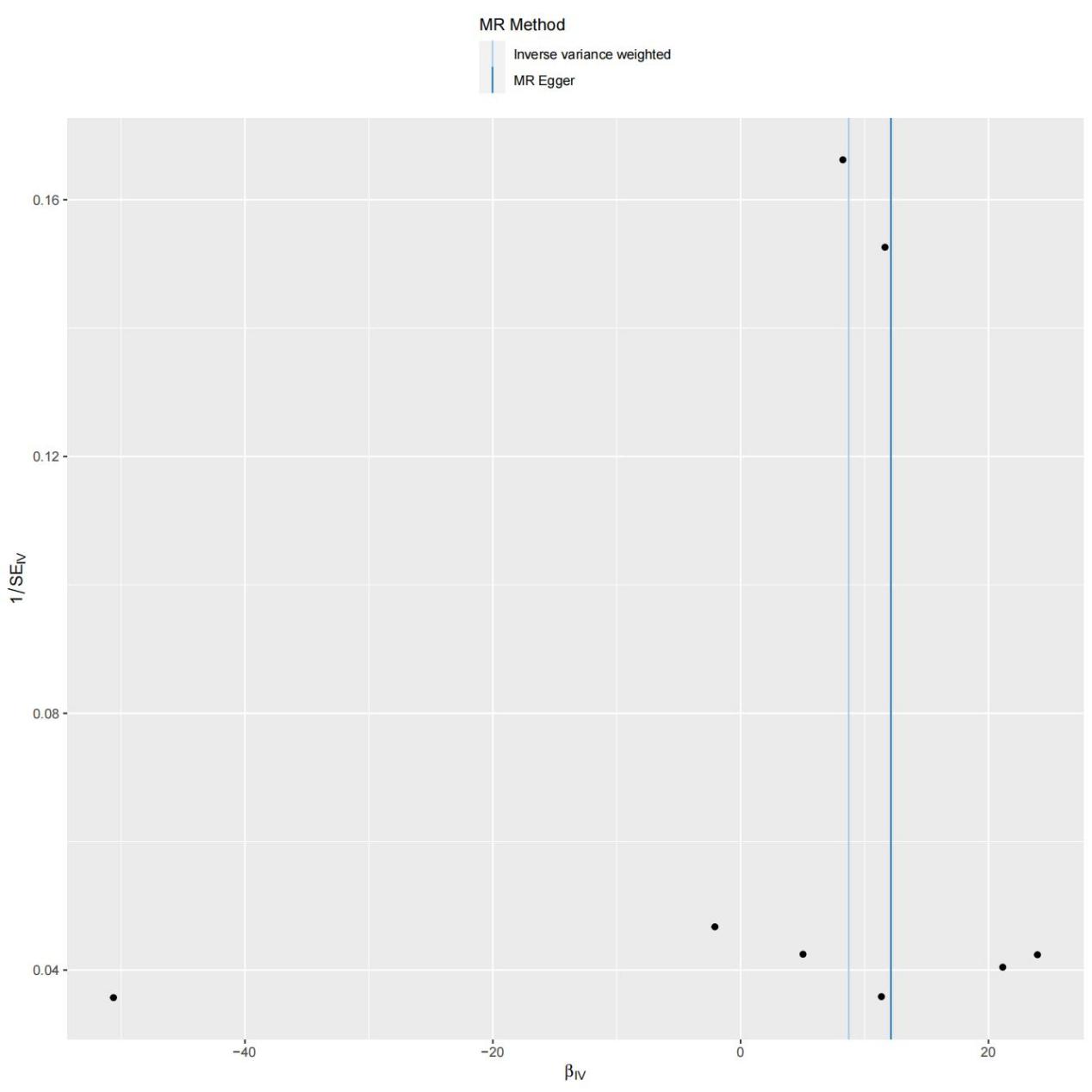

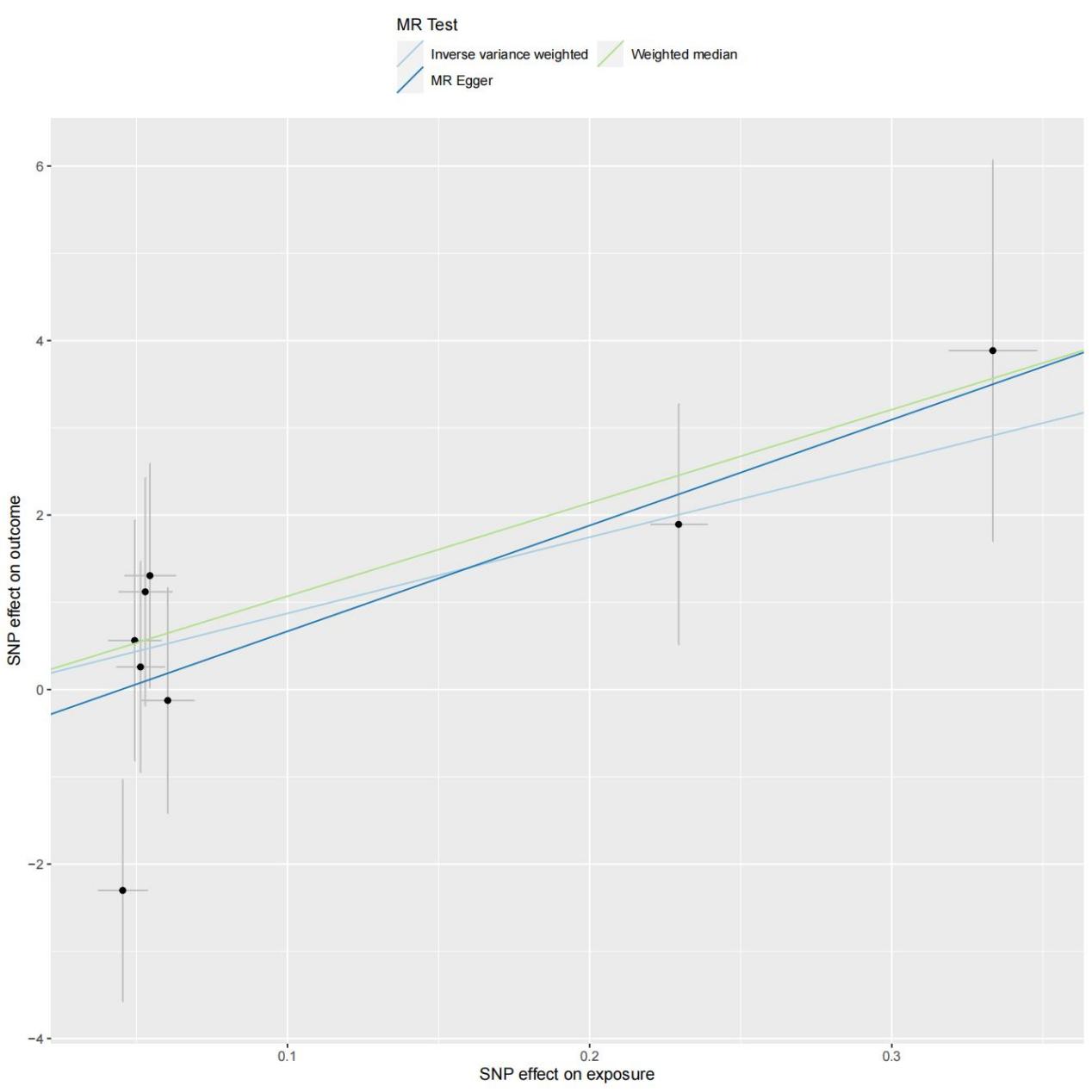
**

**d**

**
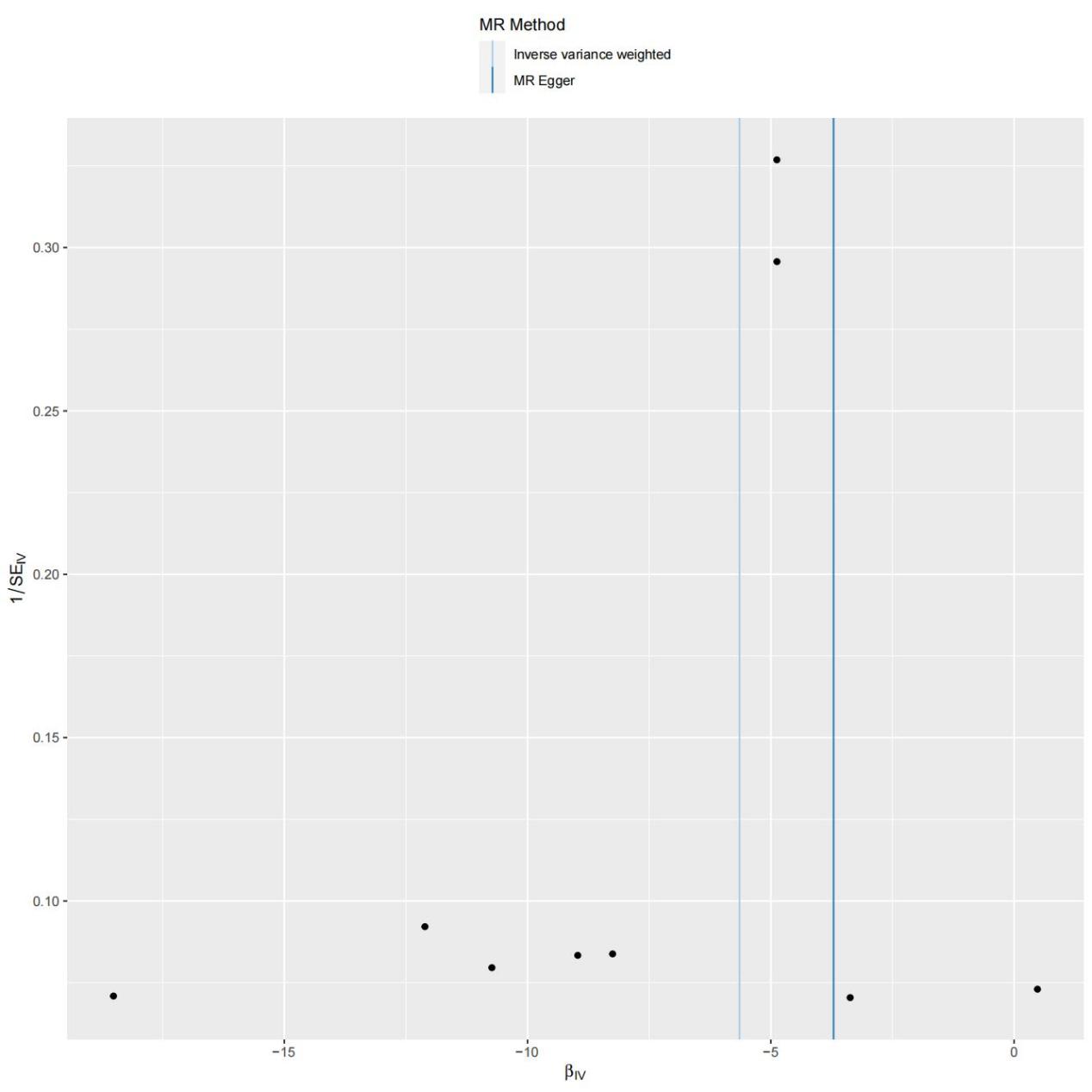

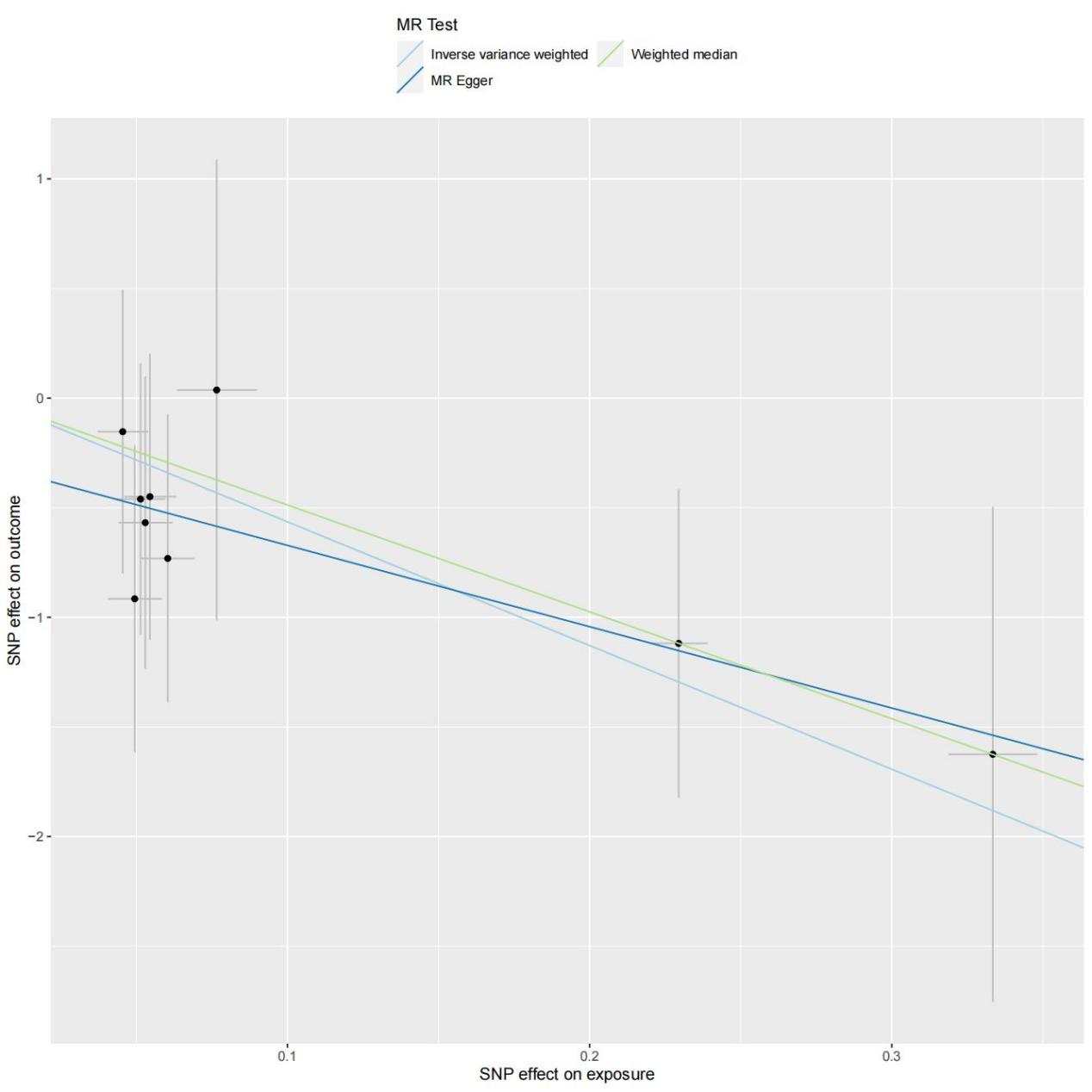
**

**e**

**
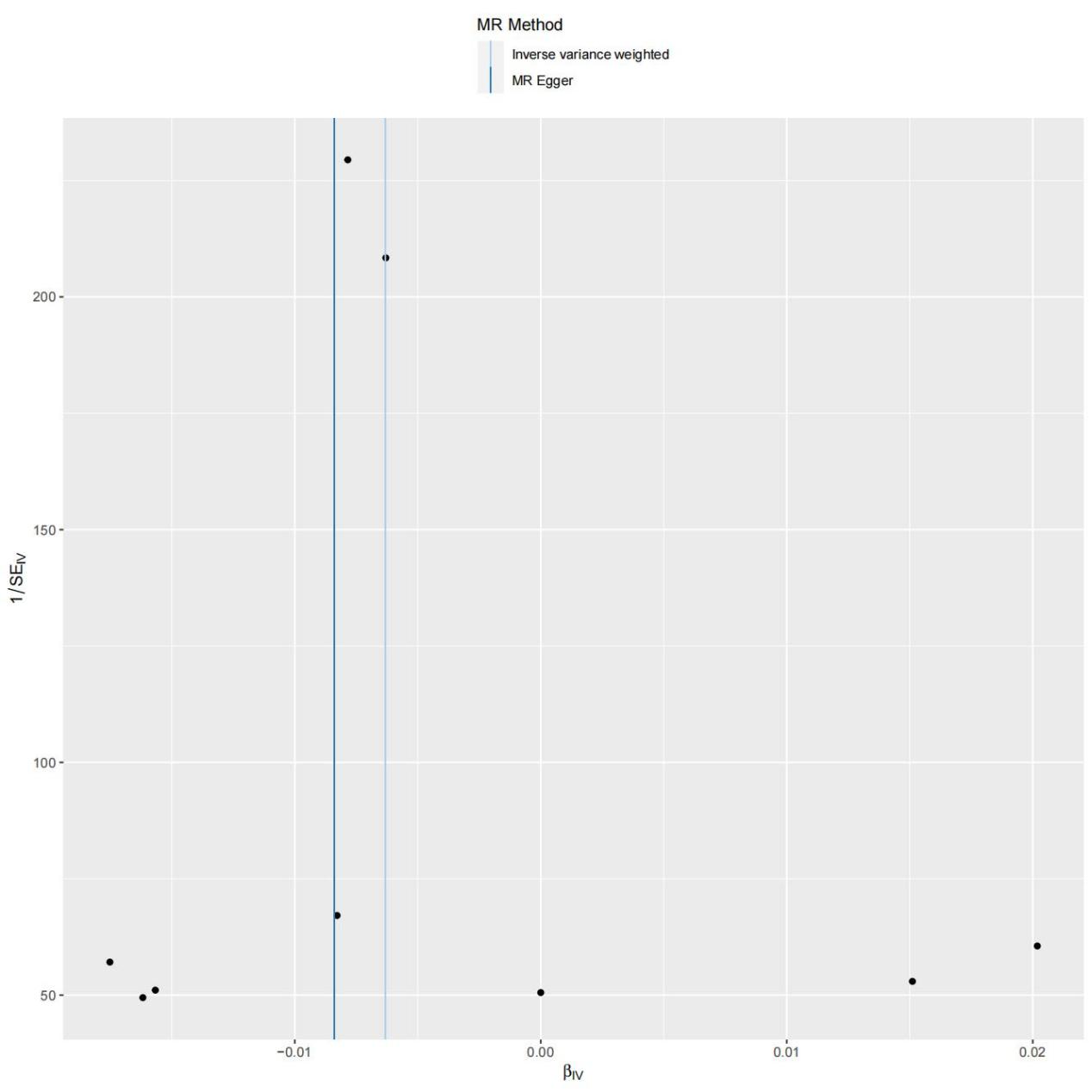

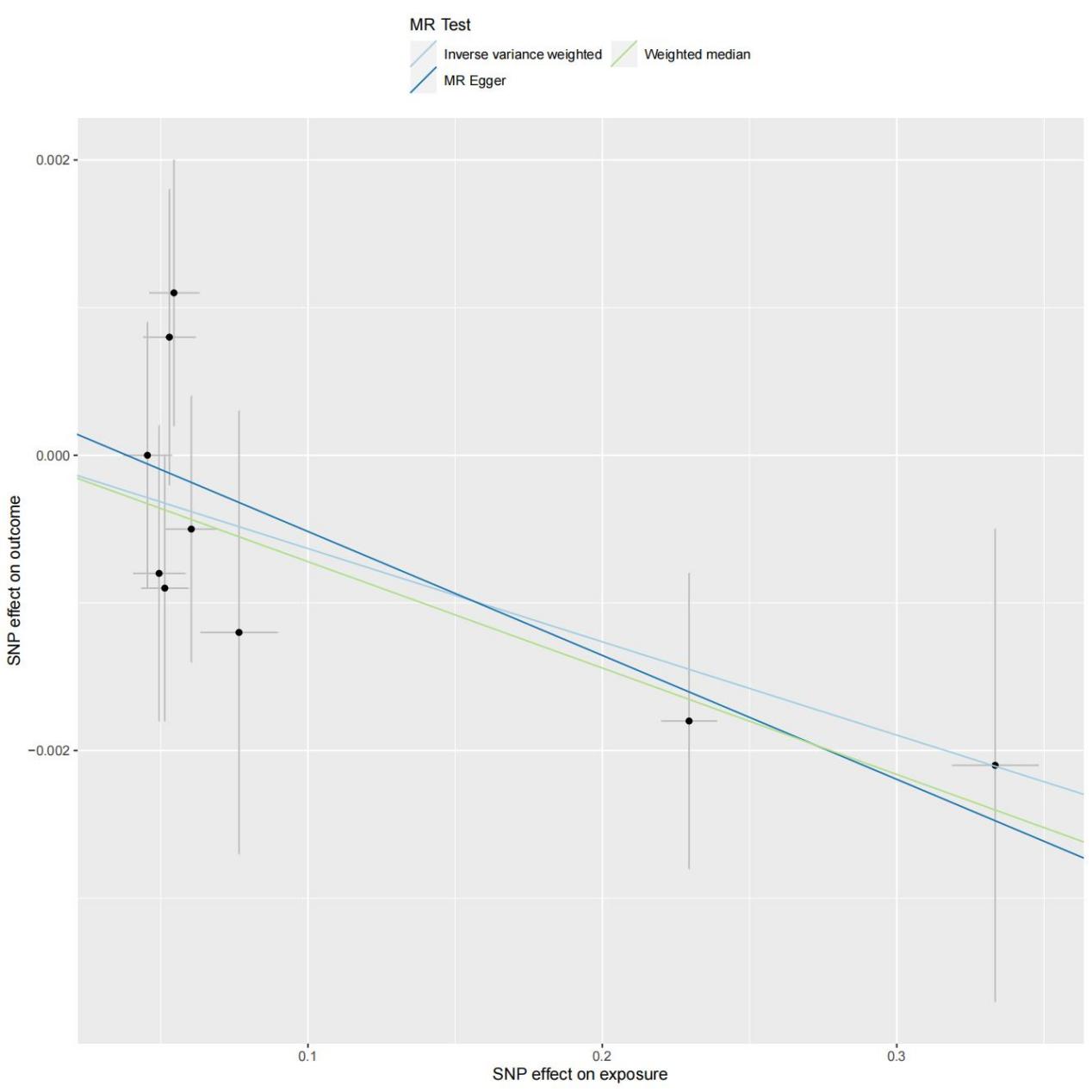
**

**f**

**
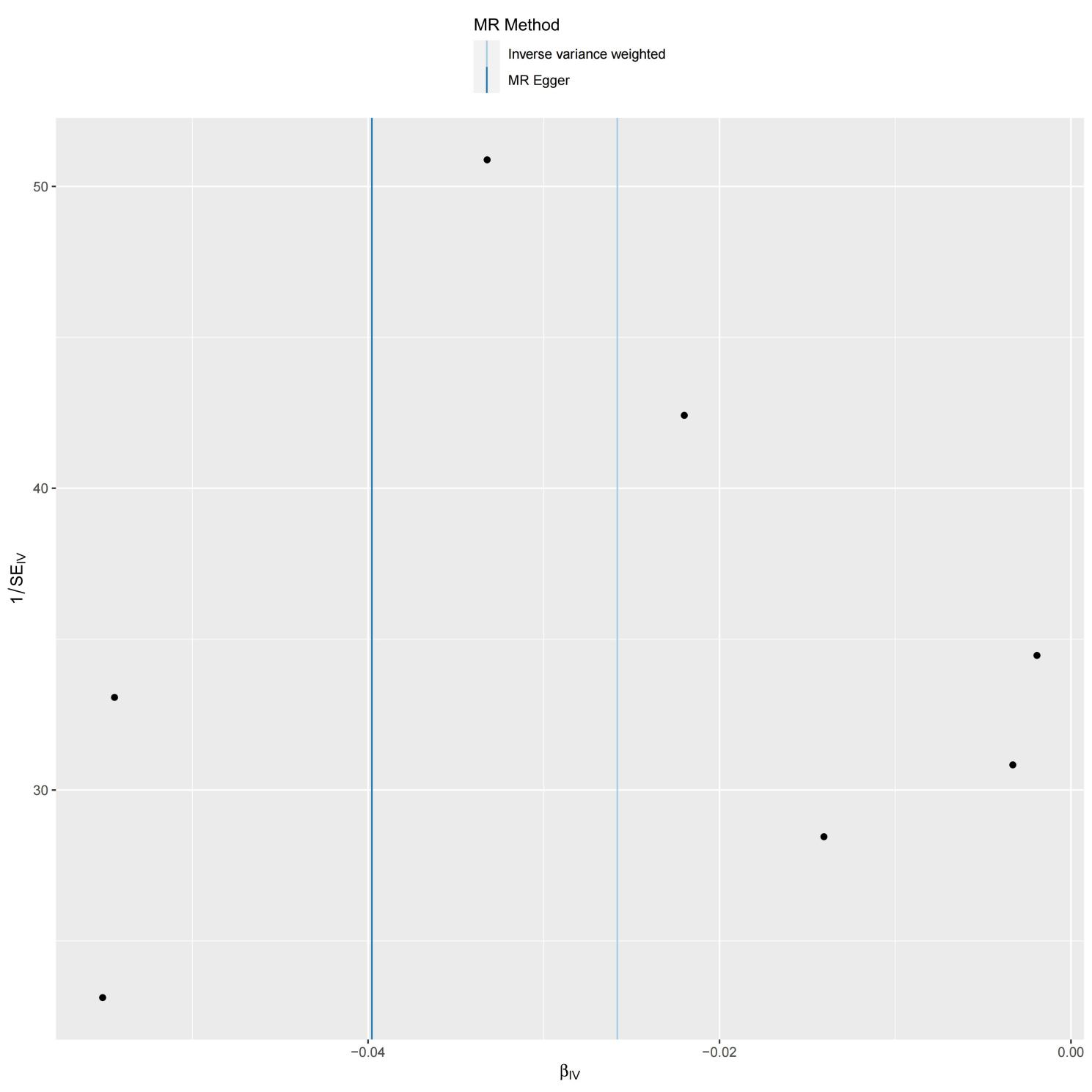

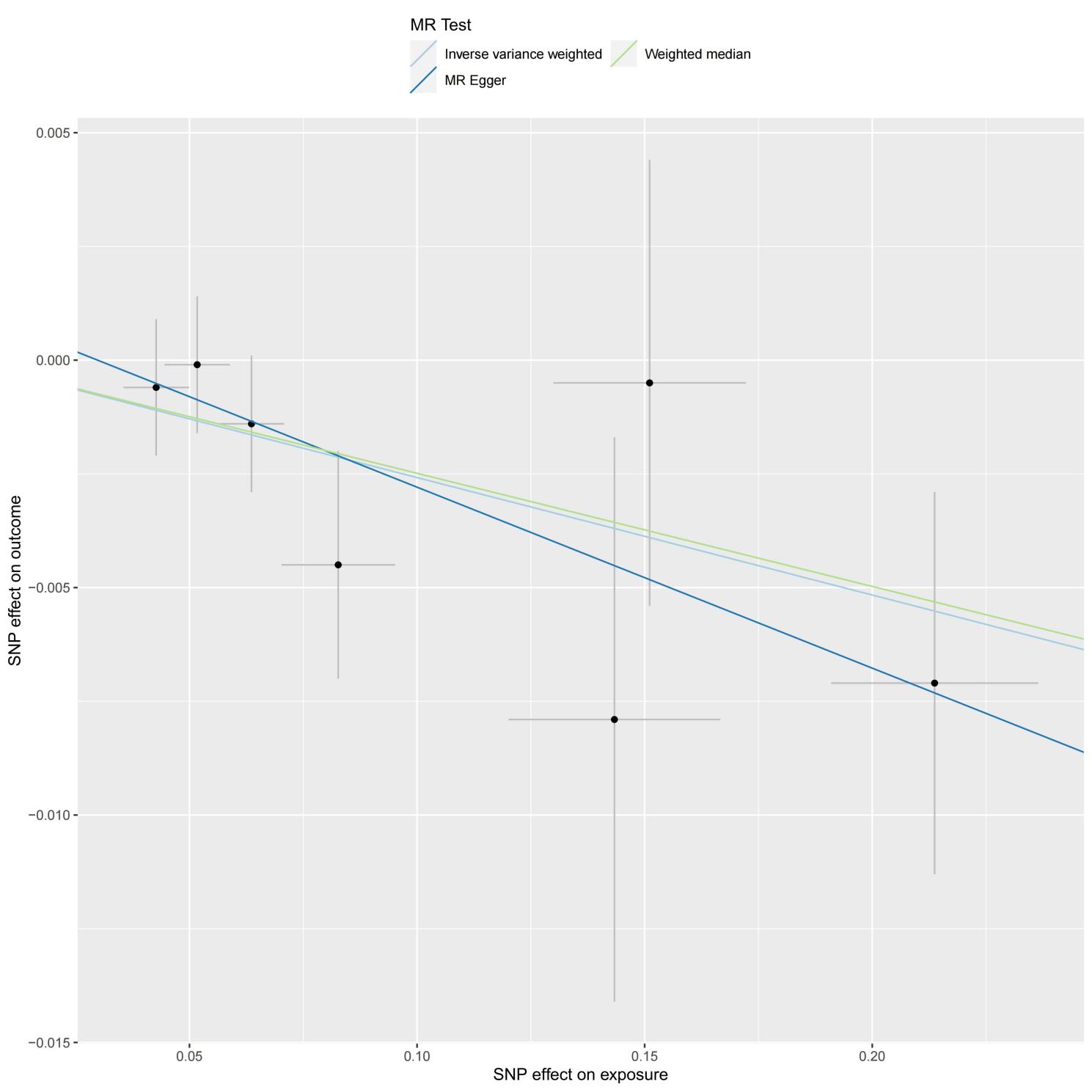
**

**g
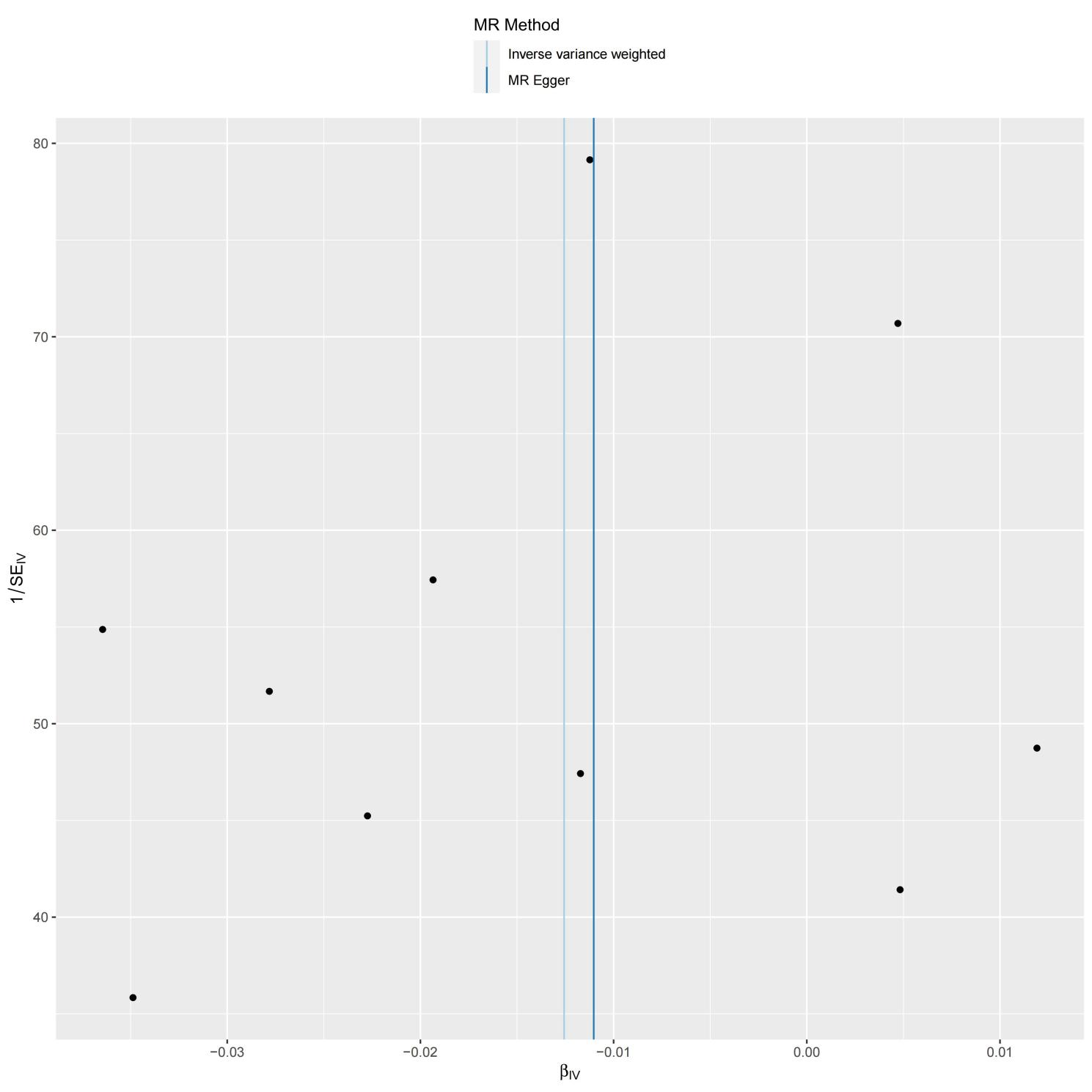

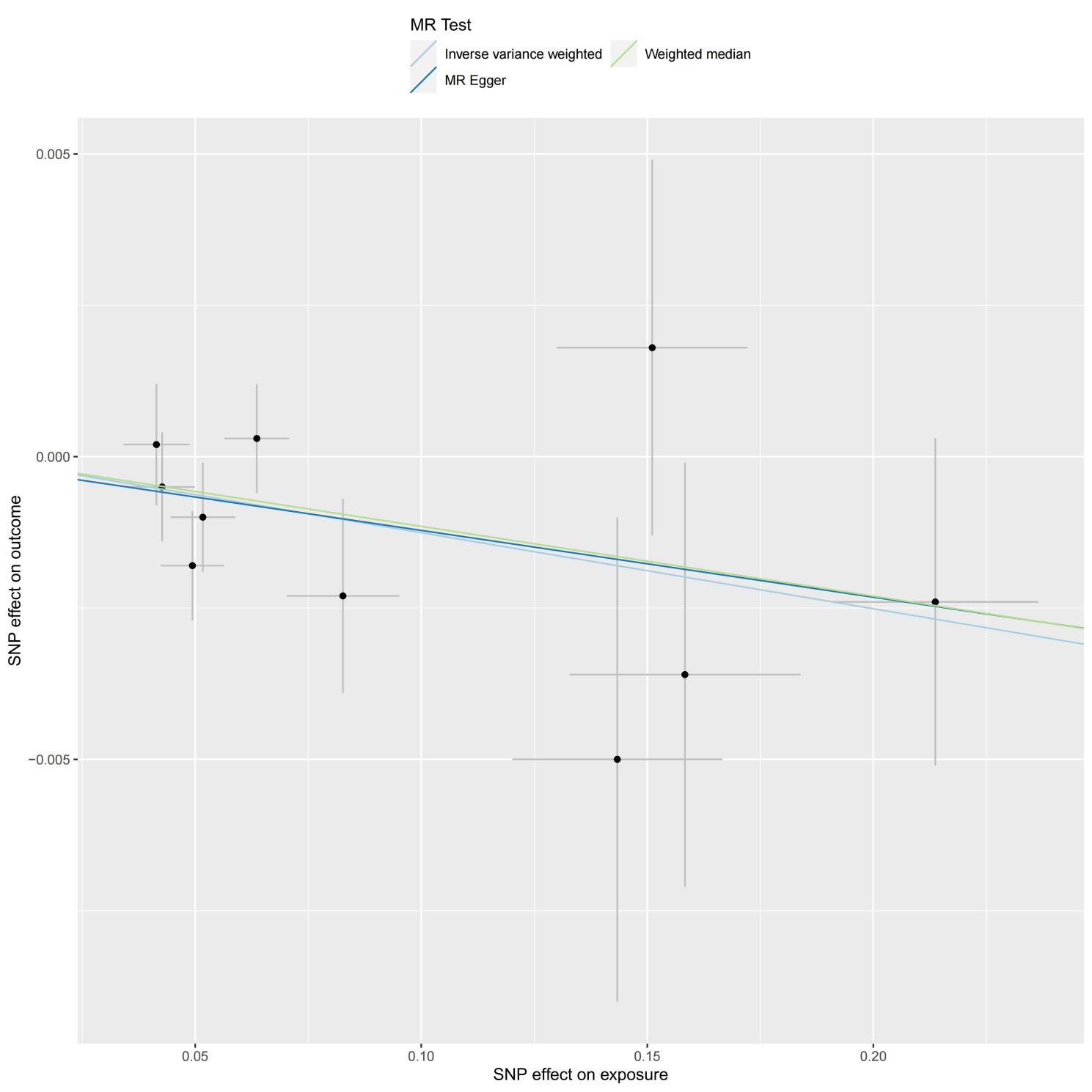
**

**h
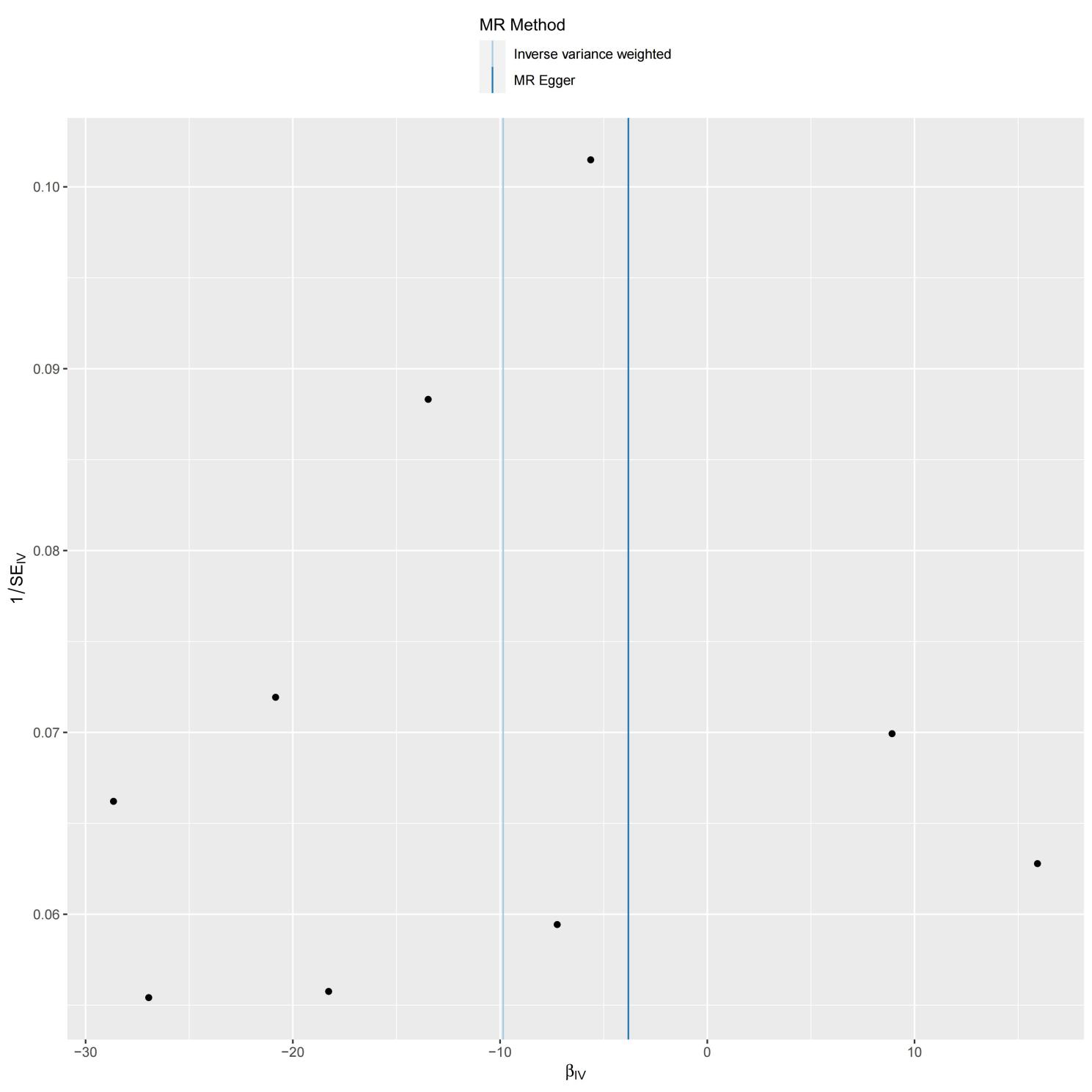

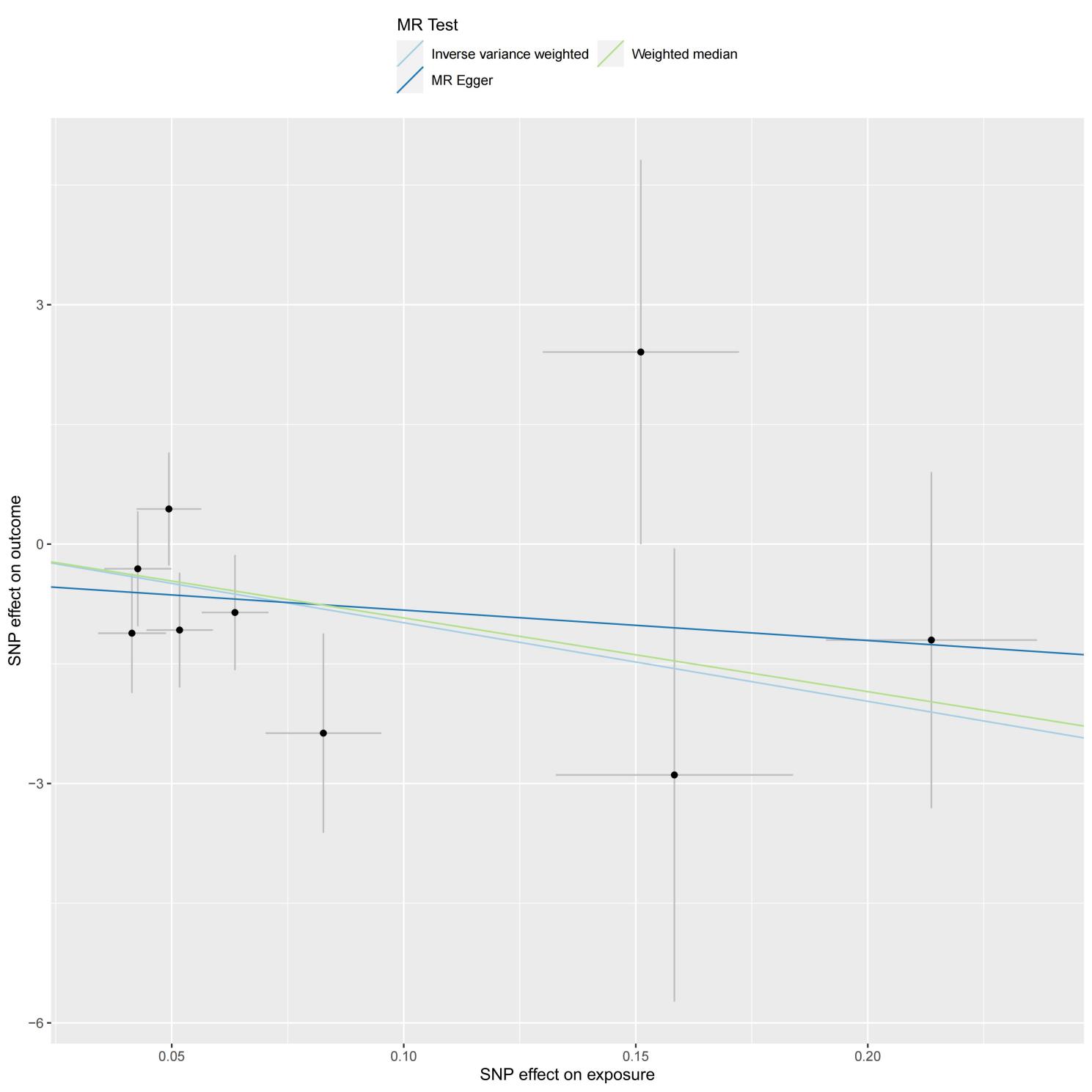
**

**i
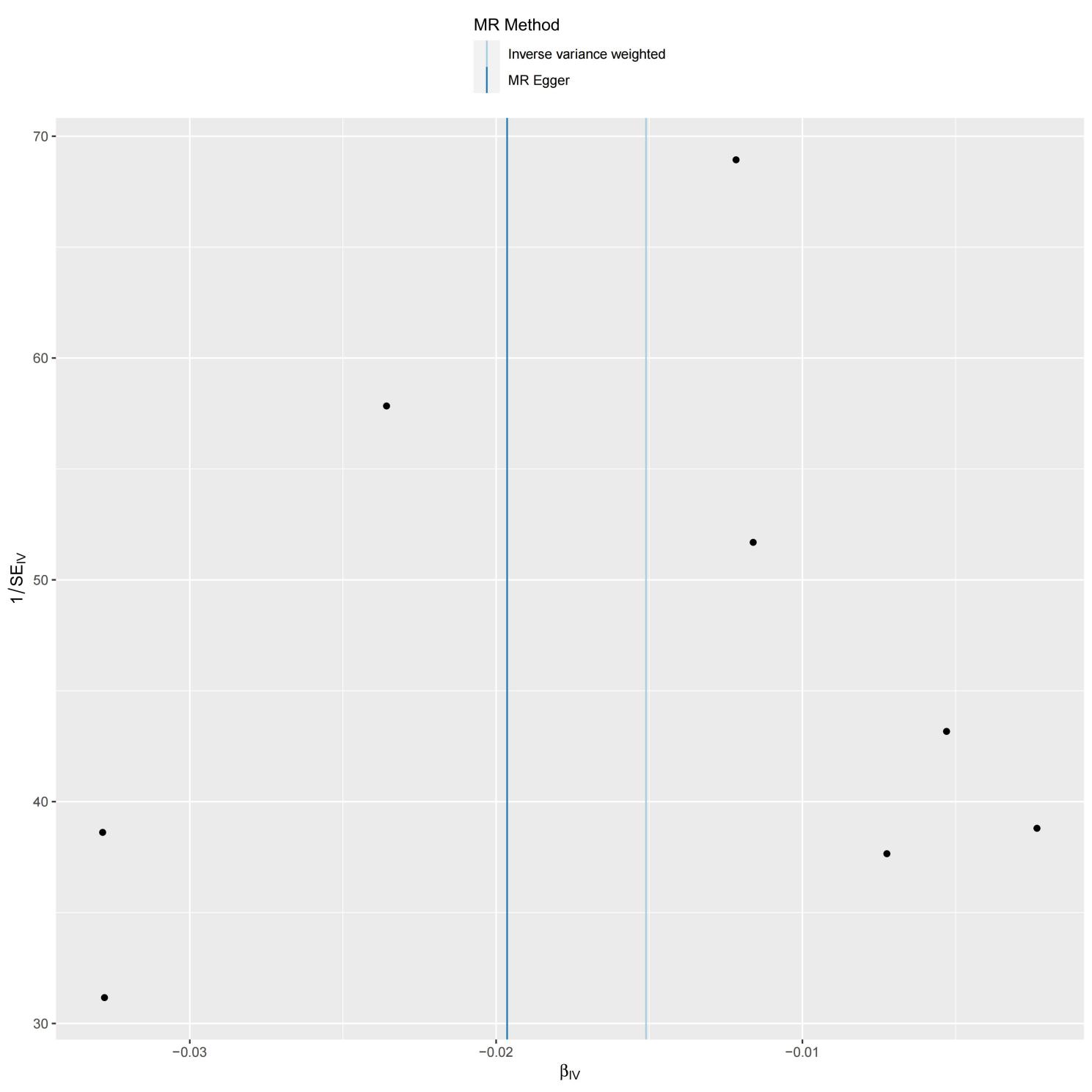

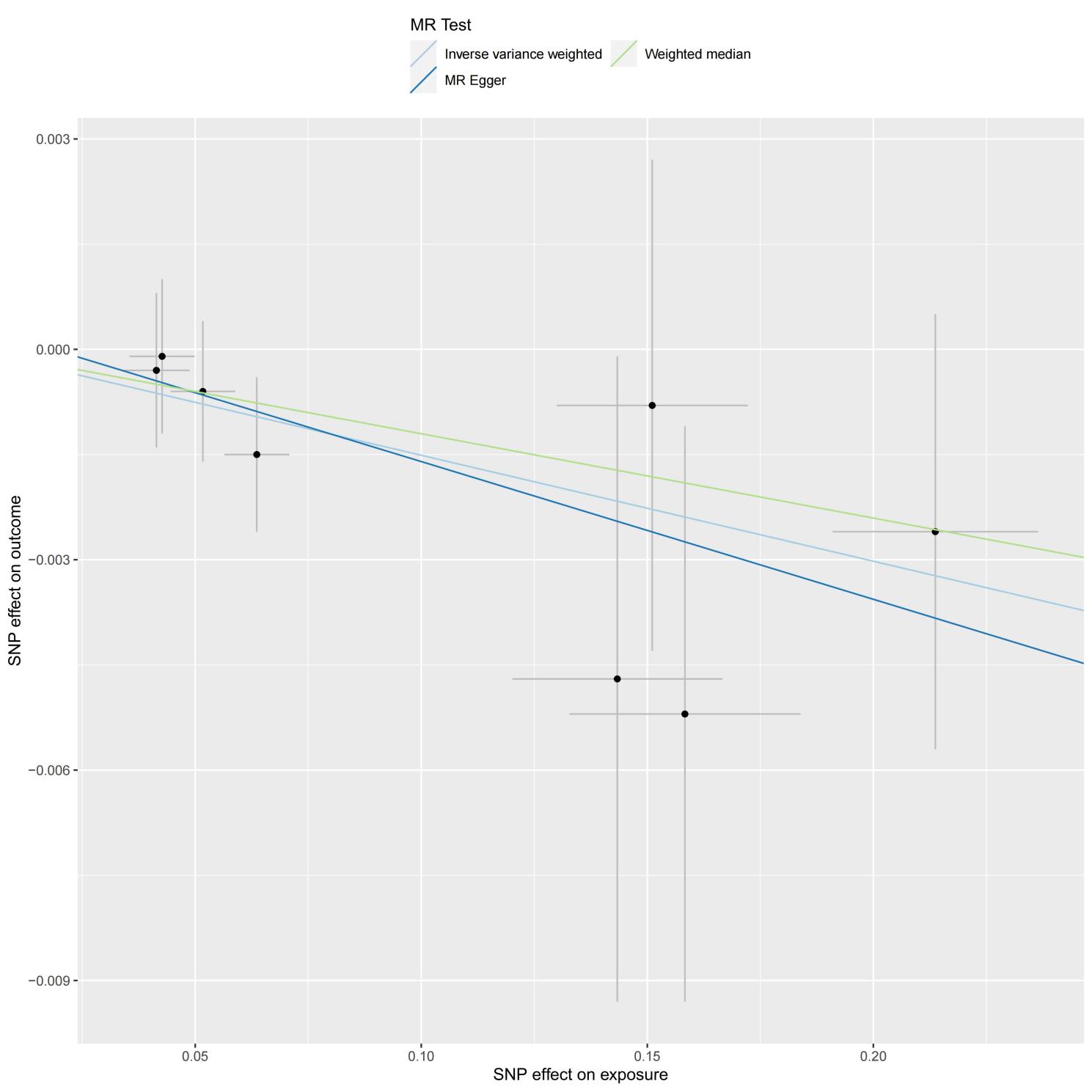
**

**j
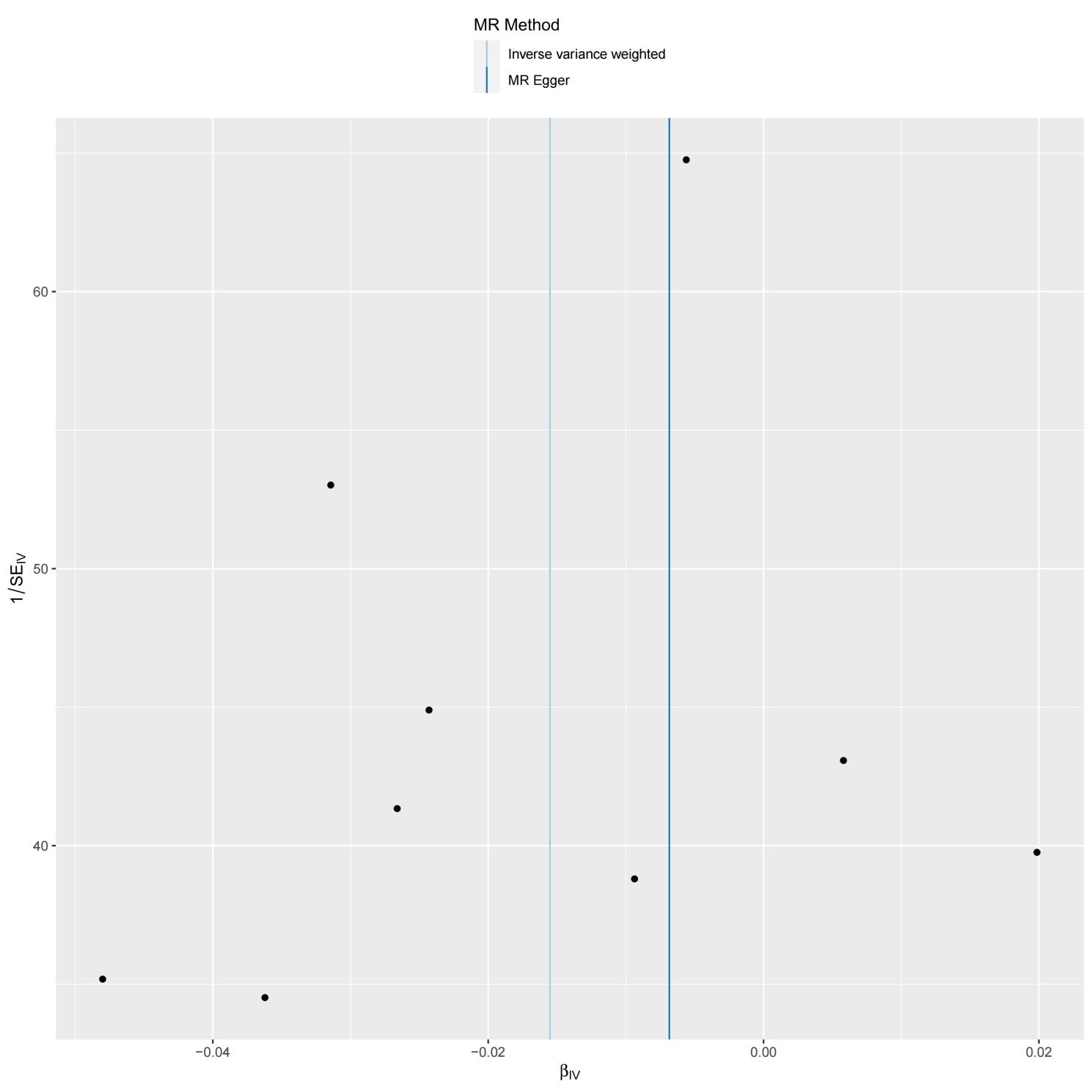

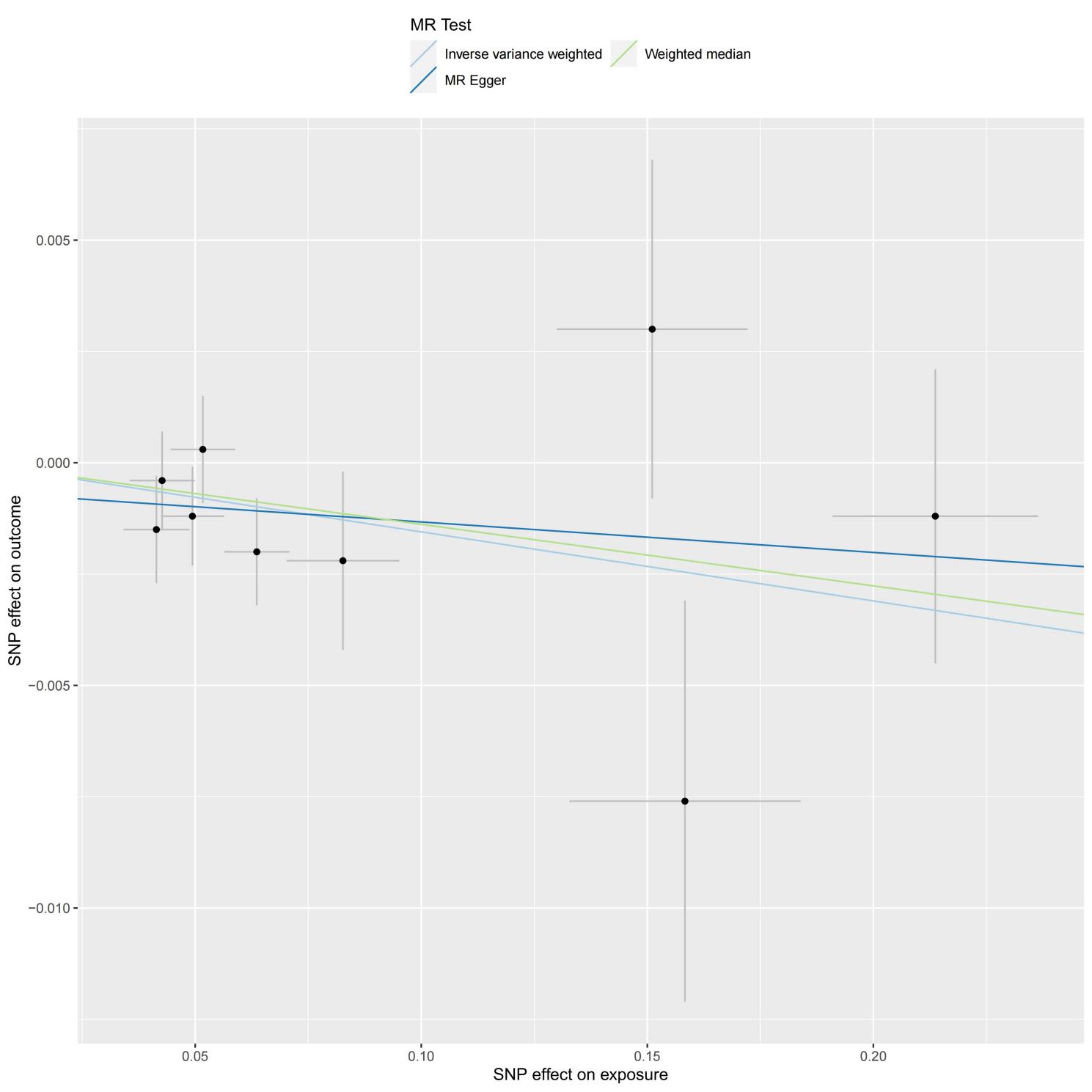
**

**k**

**
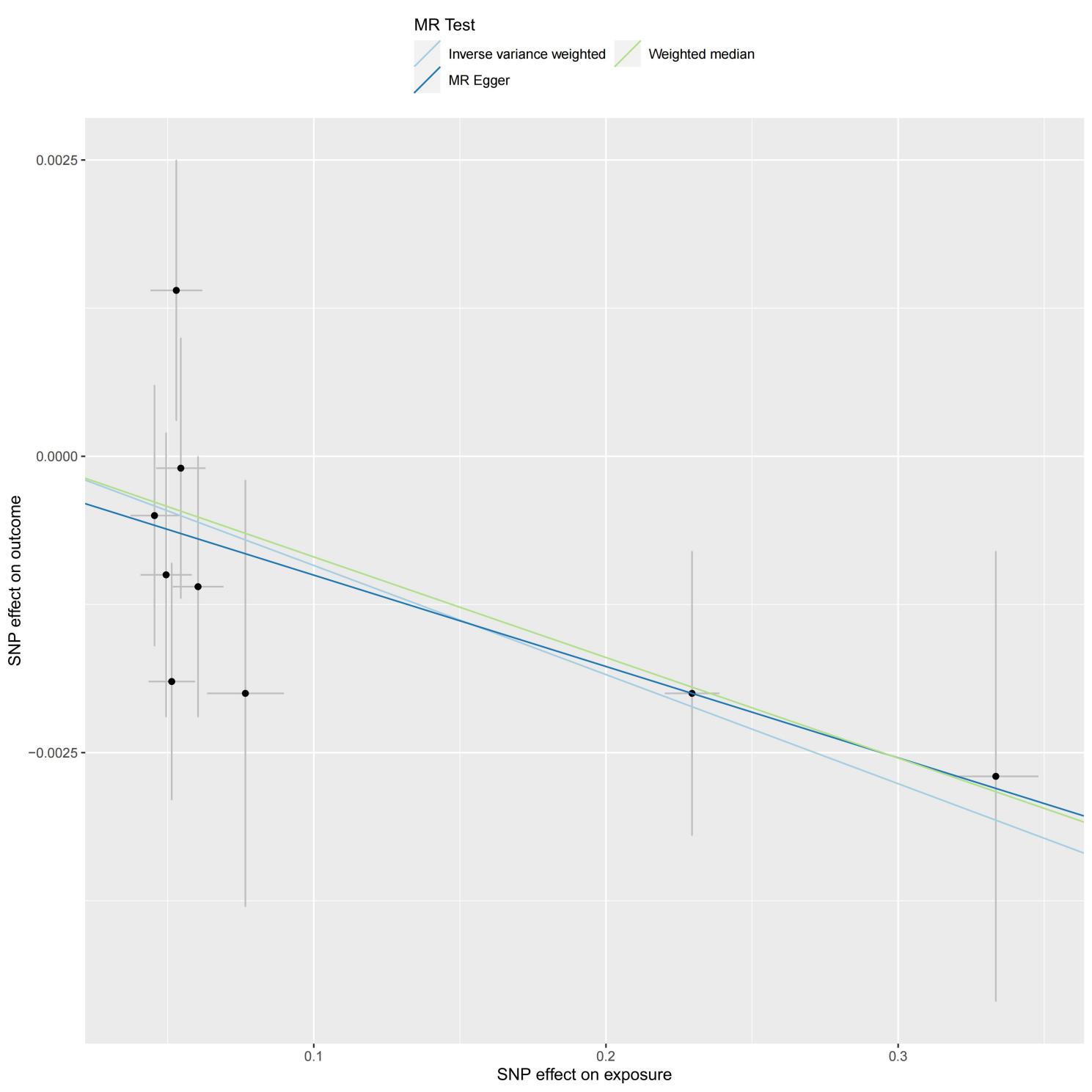

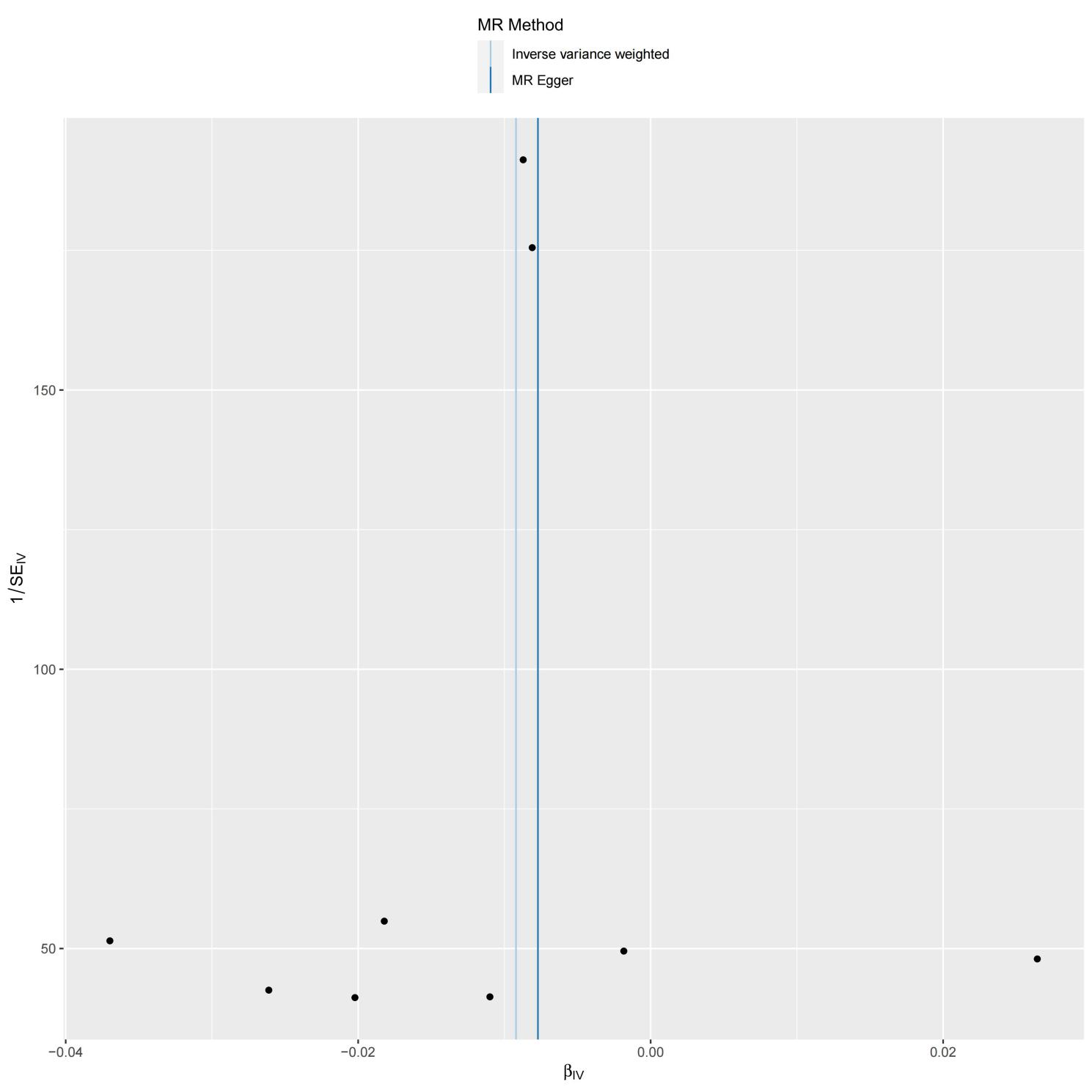
**

**l
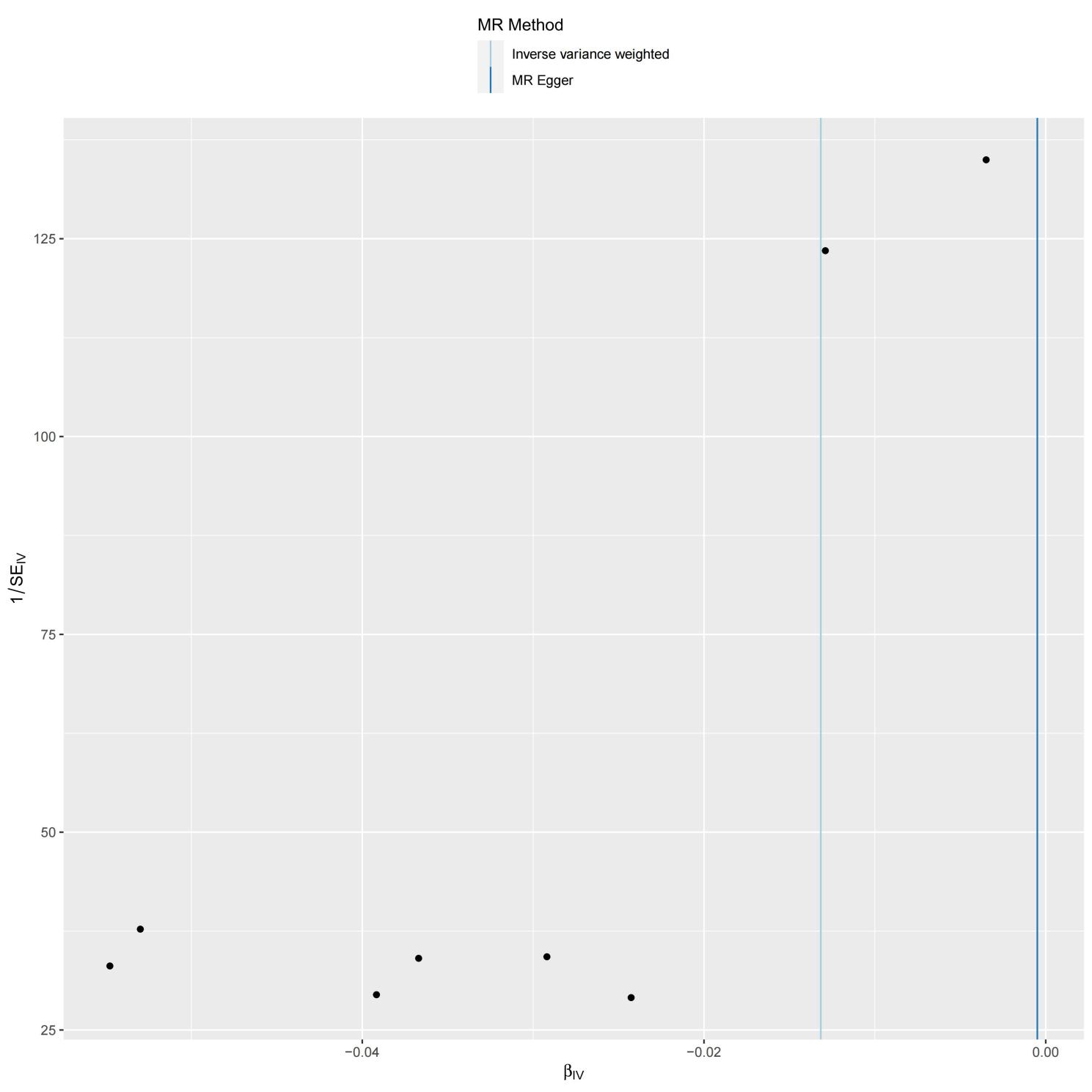

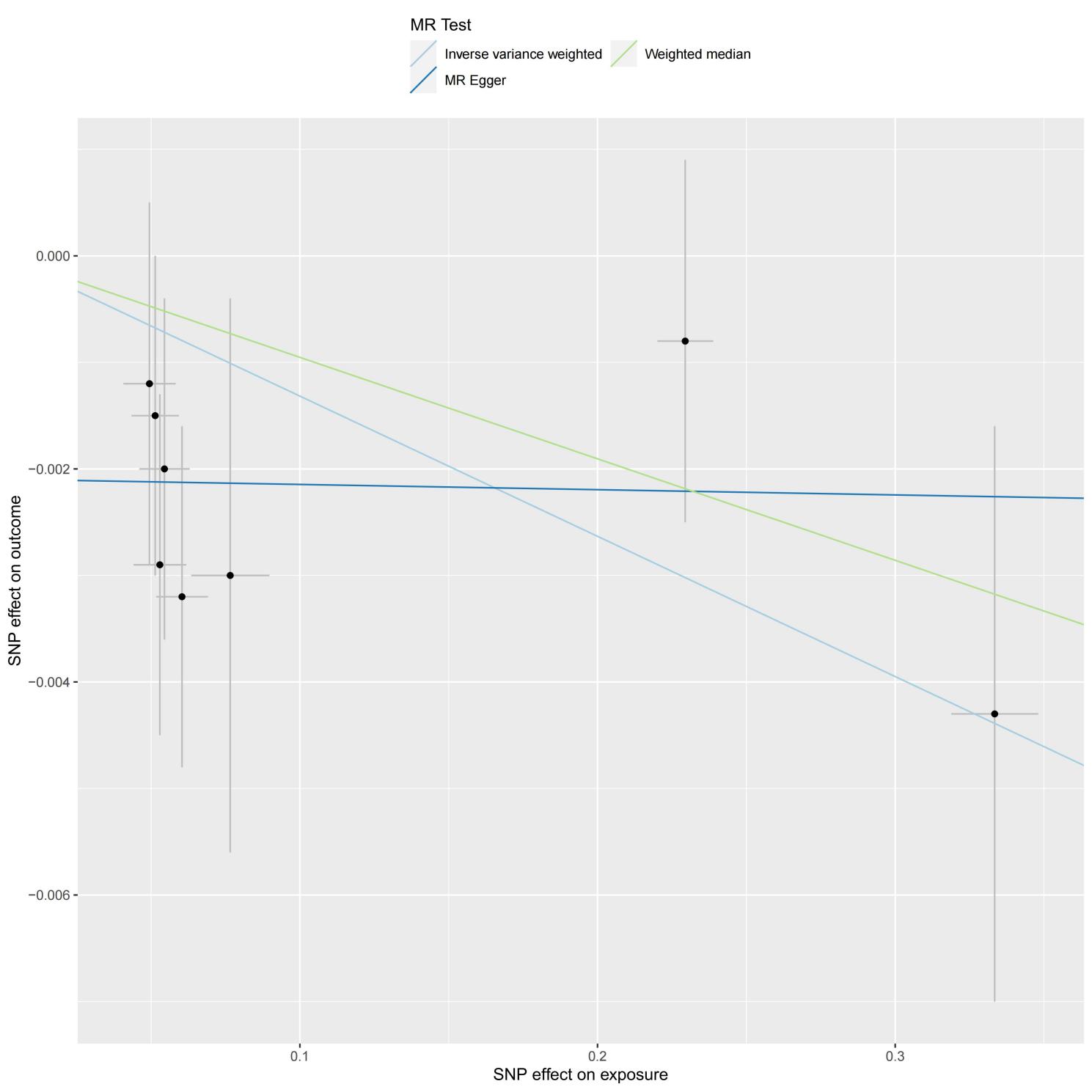
**

**m
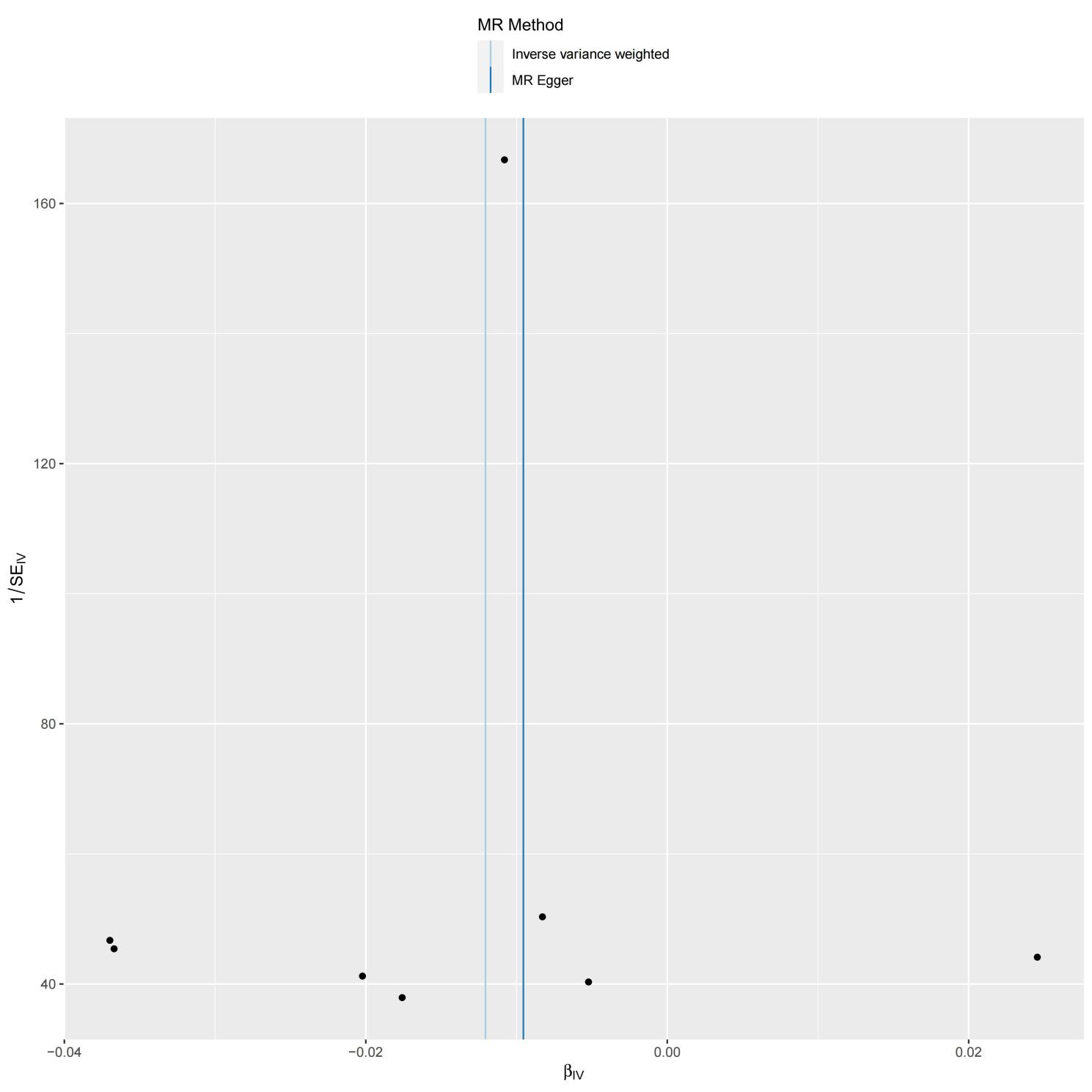

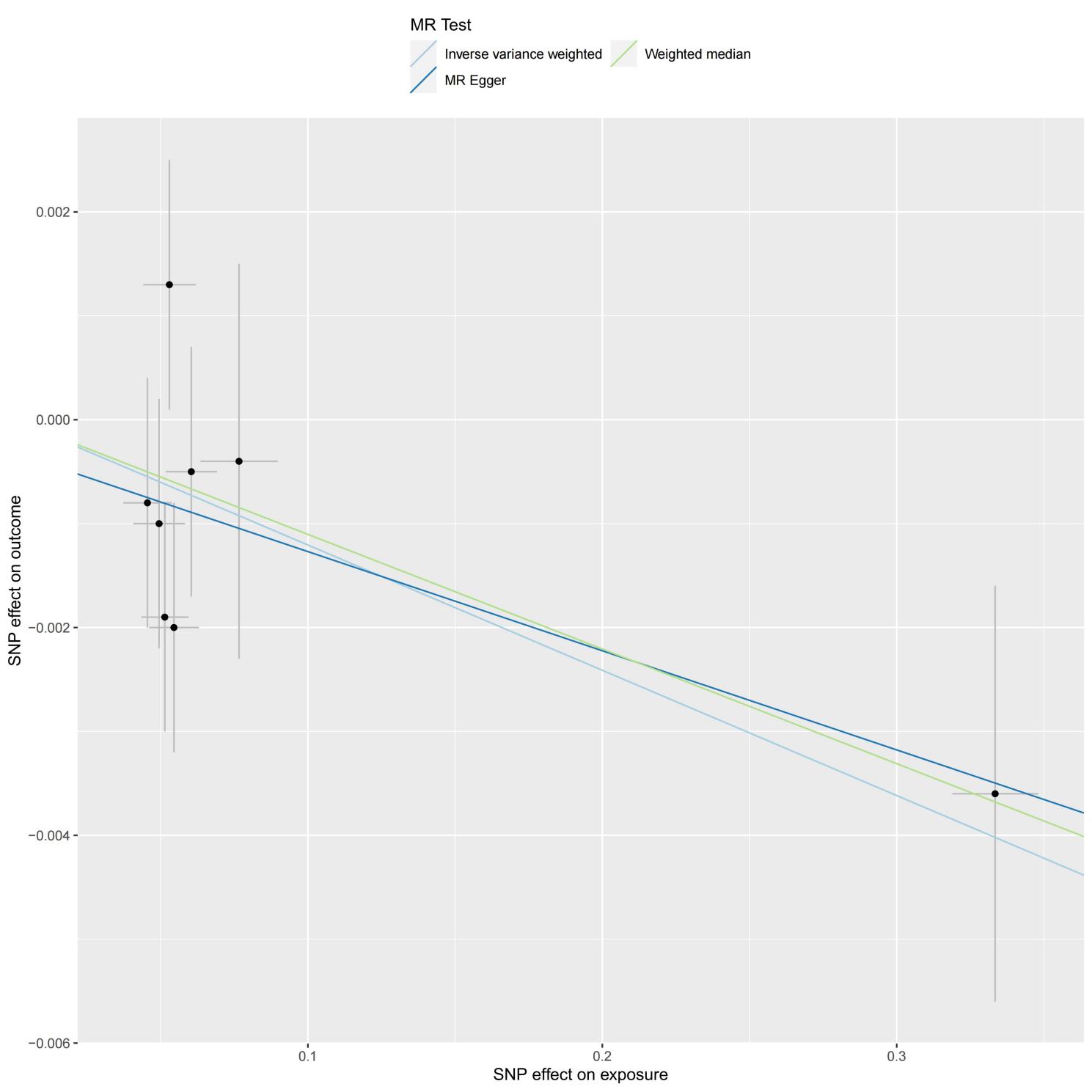
**

**n
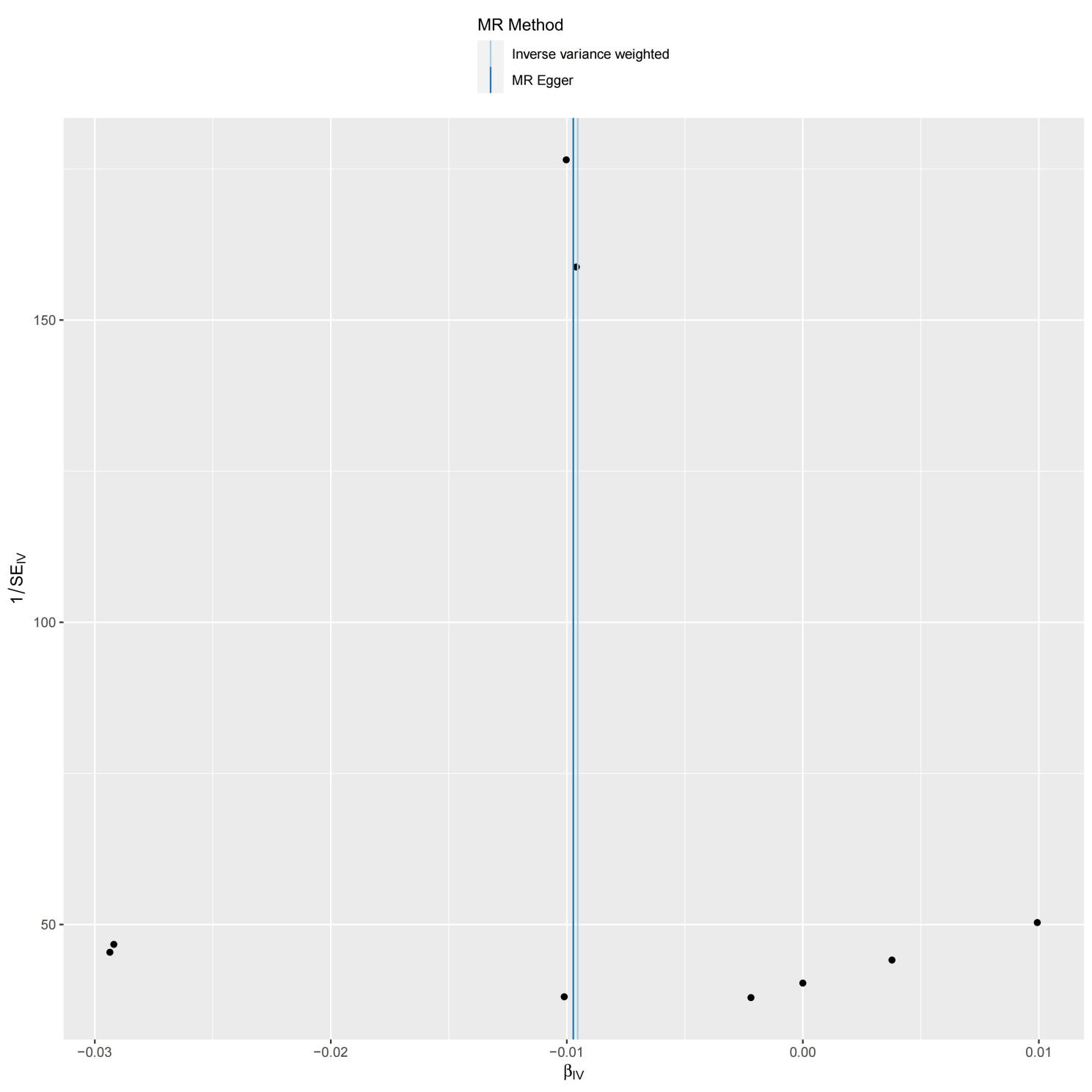

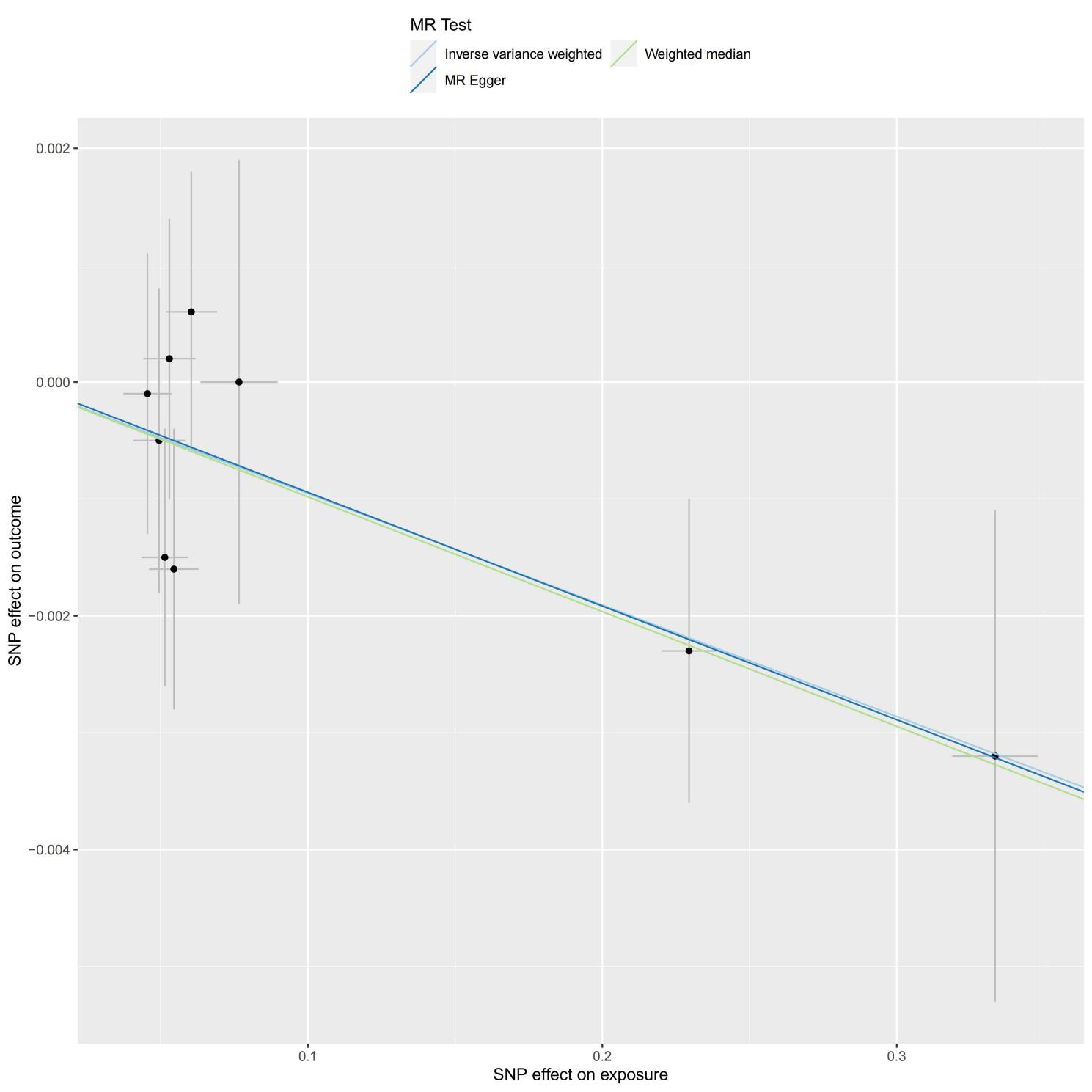
**

**o
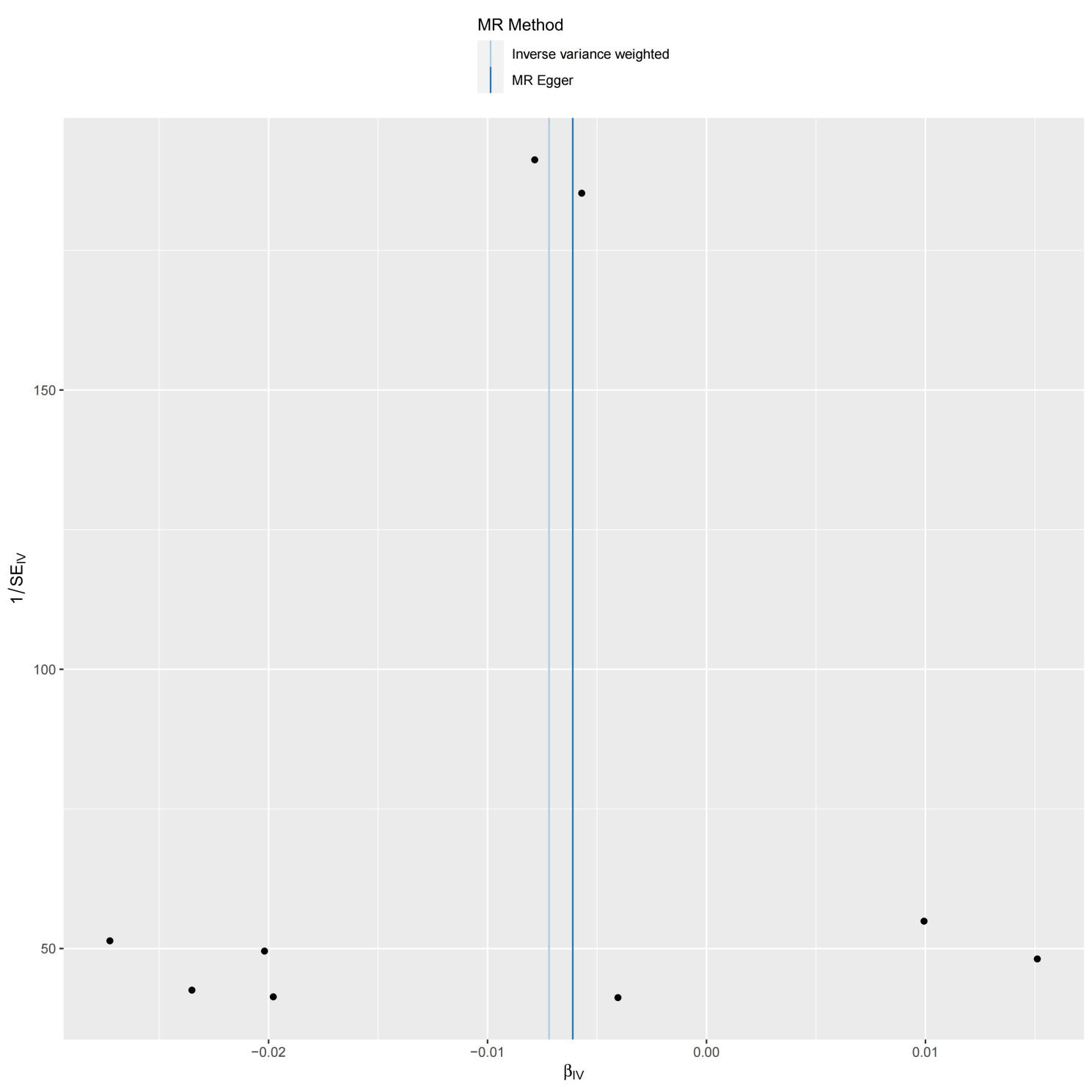

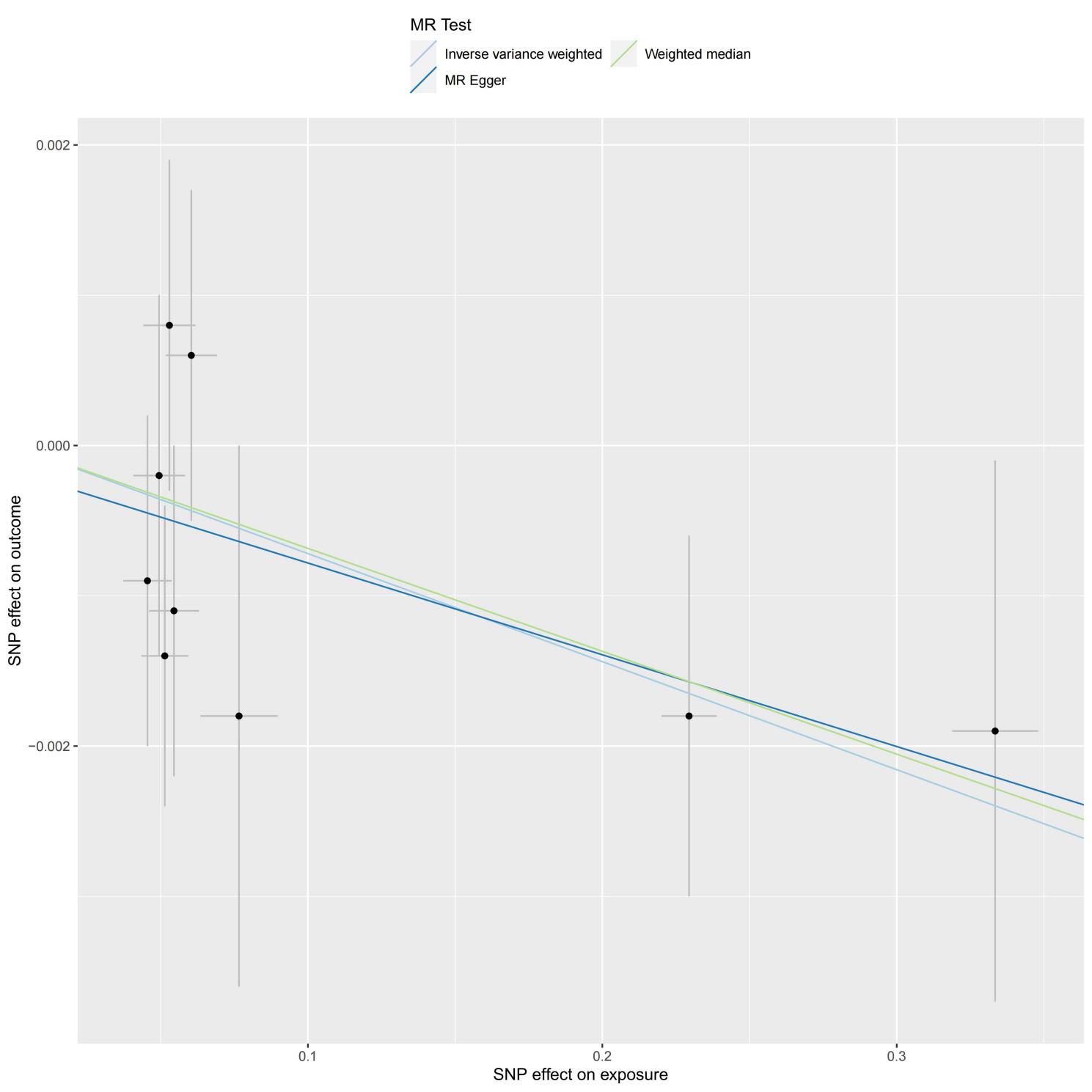
**

**p
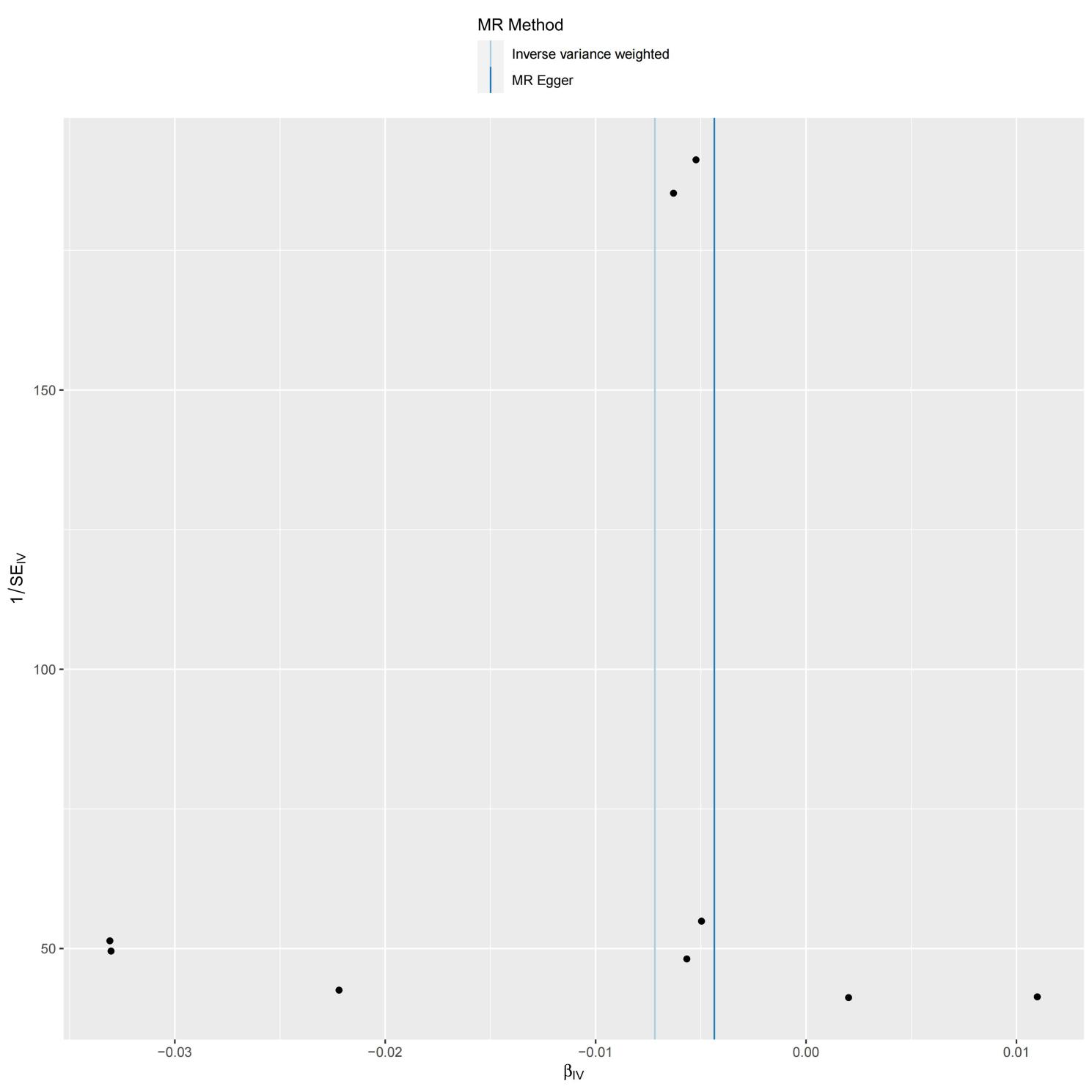

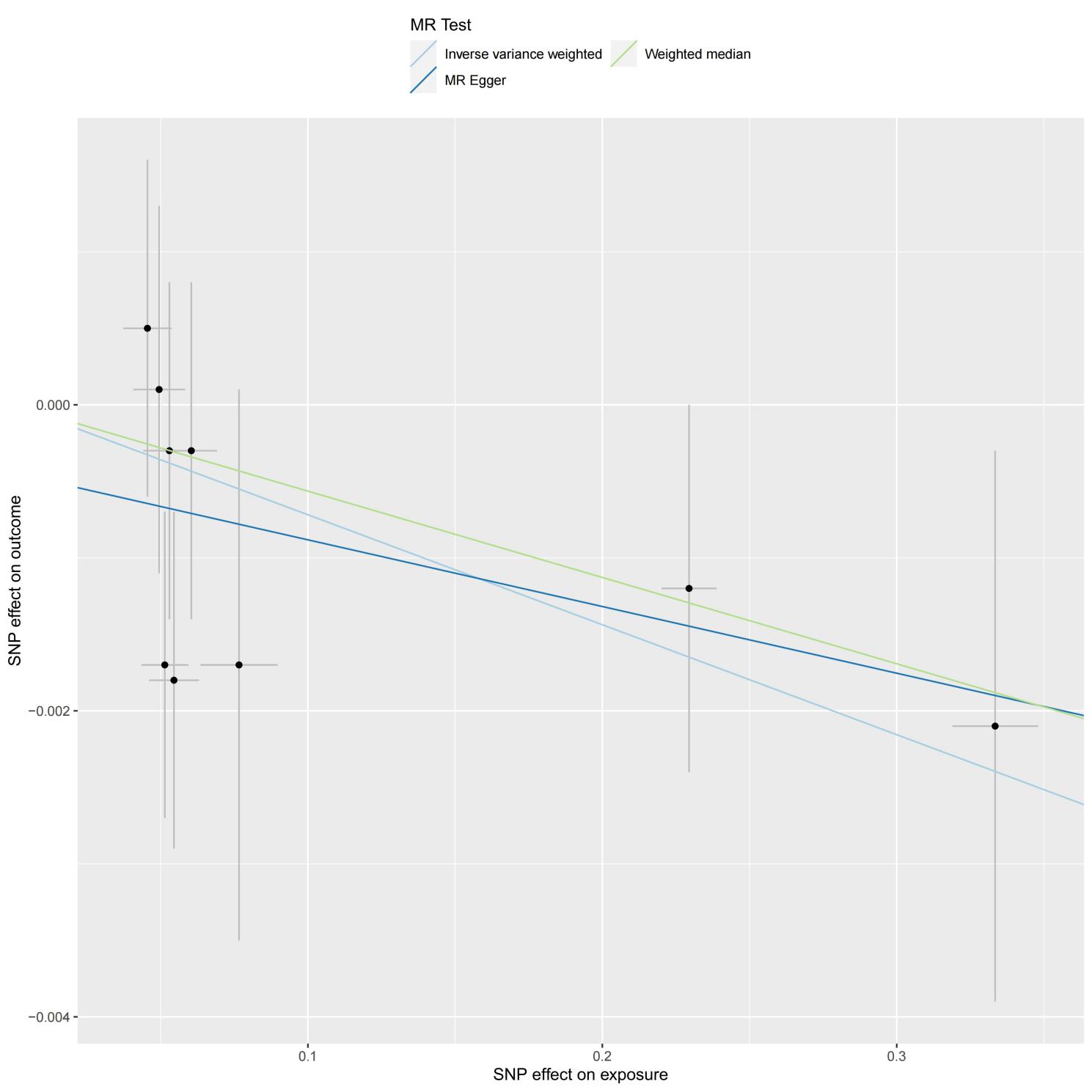
**

**q**

**Supplementary Figure S1.** Scatter plots and Funnel plots of significant estimates from (a) genetically predicted liver volume on with global weighting isthmuscingulate TH; (b)genetically predicted liver volume on with global weighting parahippocampal TH; (c) genetically predicted liver volume on without global weighting parsopercularis TH; (d) genetically predicted liver fat on without global weighting cenues SA; (e) genetically predicted liver fat on with global weighting parahippocampal SA (f) genetically predicted liver fat on with global weighting cuneus TH; (g) genetically predicted liver volume on with non-global weighting isthmuscingulate TH; (h) genetically predicted liver volume on with non-global weighting lingual TH; (i) genetically predicted liver volume on with non-global weighting parahippocampal SA; (j) genetically predicted liver volume on with non-global weighting parsopercularis TH; (k) genetically predicted liver volume on with non-global weighting precentral TH; (l) genetically predicted liver fat on with non-global weighting cuneus TH; (m) genetically predicted liver fat on with non-global weighting isthmuscingulate TH; (n) genetically predicted liver fat on with non-global weighting parsopercularis TH; (o) genetically predicted liver fat on with non-global weighting parstriangularis TH; (p) genetically predicted liver fat on with non-global weighting rostralmiddlefrontal TH; (q) genetically predicted liver fat on with non-global weighting superiorparietal SA.
